# Stealth replication of SARS-CoV-2 Omicron in the nasal epithelium at physiological temperature

**DOI:** 10.1128/jvi.02008-25

**Published:** 2025-12-19

**Authors:** Bárbara F. Fonseca, Rémy Robinot, Vincent Michel, Akram Mendez, Samuel Lebourgeois, Chloé Chivé, Raphaël Jeger-Madiot, Roshan Vaid, Vincent Bondet, Elizabeth Maloney, Florence Guivel-Benhassine, Olivier Schwartz, Darragh Duffy, Tanmoy Mondal, Samy Gobaa, Lisa A. Chakrabarti

**Affiliations:** 1 Control of Chronic Viral Infections Group, Virus & Immunity Unit, Institut Pasteur, Université Paris Cité 27058, Paris, France; 2 Biomaterials and Microfluidics core facility, Institut Pasteur, Université Paris Cité 27058, Paris, France; 3 Pathogenesis of Vascular Infections Unit, Institut Pasteur, Université Paris Cité 27058, Paris, France; 4 Department of Laboratory Medicine, Institute of Biomedicine, University of Gothenburg 3570, Gothenburg, Sweden; 5 Translational Immunology Unit, Institut Pasteur, Université Paris Cité 27058, Paris, France; 6 Virus & Immunity Unit, Institut Pasteur, Université Paris Cité 27058, Paris, France; 7 Department of Clinical Chemistry, Sahlgrenska University Hospital 56749, Gothenburg, Västra Götaland, Sweden; Loyola University Chicago - Health Sciences Campus, Maywood, Illinois, USA

**Keywords:** mucosal immunity, motile cilia, interferon, nasal epithelium, SARS-CoV-2

## Abstract

The COVID-19 pandemic was marked by successive waves of SARS-CoV-2 variants with distinct properties. The Omicron variant that emerged in late 2021 showed a major antigenic shift and rapidly spread worldwide. Since then, Omicron-derived variants have maintained their global dominance, for reasons that remain incompletely understood. We report that the original Omicron variant BA.1 evolved several traits that converged in facilitating viral spread. First, Omicron displayed an early replicative advantage over previous variants when grown in a reconstructed human nasal epithelium model. The increase in Omicron replication was more marked at the physiologically relevant temperature of 33°C found in human nasal passages. Omicron also caused a decrease in epithelial integrity, as measured by transepithelial electrical resistance and caspase-3 activation. Furthermore, Omicron caused a more marked loss of motile cilia at 33°C than other variants, suggesting a capacity to impair mucociliary clearance. Omicron induced a broad transcriptional downregulation of ciliary genes but only a limited upregulation of host innate defense genes at 33°C. The lower production of type I and type III interferons in epithelia infected by Omicron compared to those infected by the Delta variant, at 33°C as well as 37°C, confirmed the increased capacity of Omicron to evade the innate antiviral response. Thus, Omicron combined replication speed, motile cilia impairment, and limited induction of innate antiviral responses when propagated in nasal epithelia at physiological temperature. Omicron has the capacity to propagate rapidly but stealthily in the upper respiratory tract, which likely contributed to the evolutionary success of this SARS-CoV-2 variant.

**IMPORTANCE:**

The COVID-19 pandemic was initially characterized by a rapid succession of viral variants that emerged independently of each other, with each of these variants outcompeting the previous one. A major evolutionary shift occurred in late 2021, with the emergence of the highly divergent Omicron BA.1 variant. Since then, all the dominant SARS-CoV-2 variants have been derived from Omicron, for reasons that remain incompletely understood. Here, we compared the replication of SARS-CoV-2 variants in a human nasal epithelium model grown at 37°C and also at 33°C, a temperature that approximates that found in the nasal cavity. In this primary epithelial model, Omicron showed an early replicative advantage that was more marked at 33°C. However, Omicron triggered only a minimal antiviral interferon response at this temperature. Omicron could thus propagate rapidly while partly escaping the innate response at physiological nasal temperature, which helps account for the efficient dissemination of this variant worldwide.

## INTRODUCTION

The COVID-19 pandemic was characterized by sequential evolutionary shifts, with a rapid succession of viral variants rising to global dominance (1, 2). The initial SARS-CoV-2 Wuhan strain that emerged in late 2019 was replaced in the first semester of 2020 by a variant with higher infectivity, due to the presence of a D614G mutation in the S1 surface subunit of the Spike protein (3). Starting from late 2020, a series of variants termed Alpha, Beta, Gamma, Delta, and Omicron emerged successively and independently of each other, with each of these variants of concern (VOCs) outcompeting previous variants. The Delta variant, which reached worldwide dominance in spring 2021, was associated with increased disease severity, which was in part ascribed to a higher fusogenic capacity of its Spike glycoprotein (4–6). A major evolutionary shift occurred in late 2021, with the emergence of the Omicron BA.1 variant that differed by more than 32 amino acids in the Spike glycoproteins from previous strains (7). Since then, all the dominant SARS-CoV-2 variants have been phylogenetically derived from Omicron, with adaptation to preexisting immunity becoming a major selective pressure for variant replacement (8, 9).

Why Omicron-derived variants have maintained their global dominance remains incompletely understood. Epidemiological studies have documented a marked increase in viral fitness upon Omicron emergence, as measured by rates of variant spread over time (10). While escape from preexisting neutralizing antibodies is thought to be a key factor to explain the Omicron surge, there are signs that this variant is also intrinsically efficient at transmission from human to human (2). In particular, longitudinal analyses of nasopharyngeal swabs showed that detectable viral replication in the nasal mucosa occurs earlier for the Omicron than the Delta variant (11). Consistent with this observation, Omicron shows faster replication kinetics than previous variants in human airway explants (12) and in reconstructed epithelial cultures derived from primary human nasal cells (13–15).

While the Omicron variant spread efficiently, it proved slightly less pathogenic than the preceding Delta variant, as measured by hospitalization rates and pneumonia severity in individuals who were not previously vaccinated or infected by SARS-CoV-2 (16, 17). These epidemiological observations are in agreement with findings in animal models, with a lower degree of lung dysfunction induced by Omicron in infected golden Syrian hamsters, ferrets, and nonhuman primates (14, 18–20). Of note, while most animal models show an attenuated Omicron phenotype, they often do not reproduce the rapid viral replication observed in the human nasal mucosa. It is also relevant that while Omicron replicates faster than Delta in human bronchus explants, it replicates more slowly than Delta in human lung explants (12) and alveolar organoids (21, 22). This differential tropism fits with the notion of a bias toward the upper over the lower respiratory tract, possibly accounting for a lower pathogenic potential of Omicron.

Different mechanisms have been proposed to account for the increased replication capacity of Omicron in human airway epithelia, including a higher affinity of Spike for the ACE-2 receptor (23, 24) or a better interaction of Spike with the negatively charged glycocalyx at the cellular surface (25), leading to an overall better attachment of viral particles to primary ciliated epithelial cells (26). Several studies have also documented that the Omicron entry pathway differs from that of previous variants, with a lower reliance on the surface serine protease TMPRSS2 to release the fusion peptide and an expanded usage of other proteases (15, 27, 28). In addition, an improved capacity to evade the innate antiviral response has also been reported for Omicron, which could contribute to its rapid replication kinetics (29–31). To further address these questions, we systematically compared the replication of Omicron and previous variants in a reconstructed human nasal epithelium model grown at the air-liquid interface (ALI). This model recapitulates key features of the nasal mucosa, including a pseudostratified epithelium structure, the presence of diverse epithelial cell types (ciliated, goblet, and basal cells), and the competence for mucociliary clearance function (32). In addition to its barrier function, the nasal epithelium has also an air conditioning function, warming and moistening inspired air to maintain the internal milieu of the lungs (33). This results in a temperature gradient from the nares to the nasopharynx, with 31°C–34°C typically measured at the posterior end of the nasal cavity in humans (34). Therefore, we chose to compare SARS-CoV-2 variant replication at 37°C but also at 33°C, a more physiological temperature that approximates that found in the nasal cavity. We report that the Omicron replicative advantage is more marked at the temperature found in nasal passages as this variant manages to spread efficiently and induce motile cilia damage while triggering only a minimal innate interferon (IFN) response from primary epithelial cells at 33°C. These findings help account for the rapid and efficient dissemination of the Omicron variant worldwide.

## RESULTS

### The replicative advantage of Omicron in nasal epithelial cells is more marked at a physiologically relevant temperature

For infection experiments, we used reconstructed human nasal epithelia generated from pooled human donors (MucilAir, Epithelix) to minimize donor-dependent variability. Prior to infection, cultures maintained at the ALI were checked for motile cilia activity and mucus production, ensuring the proper differentiation of a pseudostratified epithelium. Infectious inocula with different SARS-CoV-2 variants were normalized based on viral RNA content and infections were monitored for 4 days.

**Fig 1:**
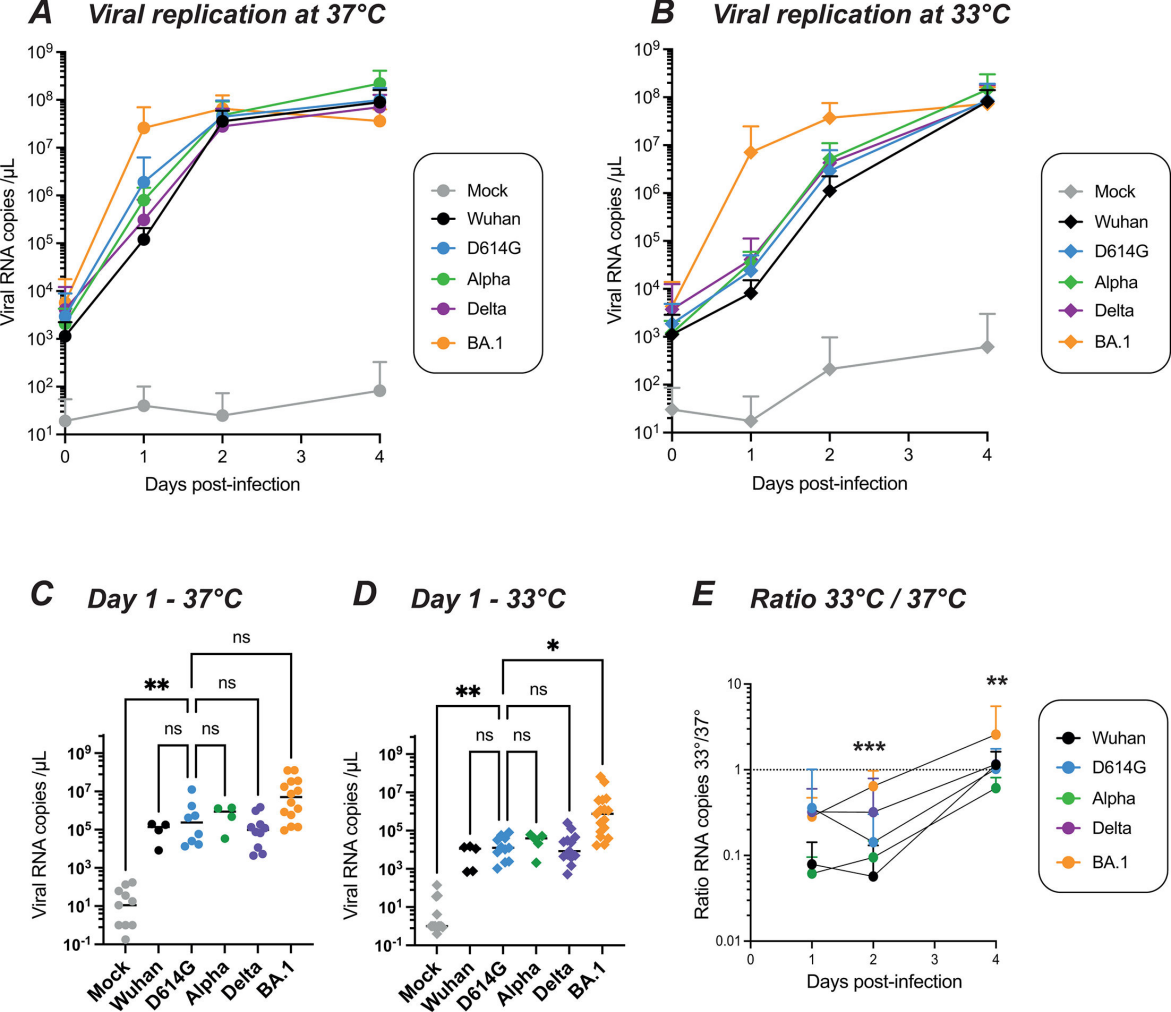
Temperature-dependent replication of SARS-CoV-2 variants in reconstructed human nasal epithelia. The viral load in apical supernatants was quantified by RT-qPCR for samples infected by the following SARS-CoV-2 lineages: Wuhan, D614G, Alpha, Delta, and Omicron BA.1. Infections were performed at an input equivalent to 10^8^ viral RNA copies. (**A, B**) Kinetics of viral replication at 37°C (**A**) and at 33°C (**B**), with means and SD reported (*n* = 4 to 14 independent samples per point). (**C,D**) Comparison of apical viral loads measured at 1 day post-infection (dpi) at 37°C (**C**) and 33°C (**D**). Medians are reported. (**E**) The ratio of the number of viral RNA copies measured at 33°C to that measured at 37°C is shown at different dpi, with means and SD reported (*n* = 4 to 14 independent samples per point).(**C, D, E**) Statistical comparisons were done between the D614G reference and all other variants or the mock condition, using the Kruskal-Wallis test with Dunn’s correction; ns: not significant; *P < 0.05; **P < 0.01; ***P < 0.001. In (**E**), the only significant differences in ratios were seen between BA.1 and D614G at 2 dpi and 4 dpi.

In nasal epithelial cultures grown at 37°C, all the viruses tested showed an efficient viral replication, including the ancestral Wuhan strain, the early variant D614G, and the later variants Alpha, Delta, and Omicron BA.1 (Fig. 1A), as well as the Beta and Gamma variants (Fig. S1A and C). Viral replication reached a plateau at day 2 (D2), except for the Omicron BA.1 variant, which reached close to plateau values at day 1 (D1). Interestingly, the replicative advantage of Omicron BA.1 became more apparent when the epithelial cultures were grown at 33°C, a temperature more relevant to nasal physiology (Fig. 1B ; Fig. S1B and D). Comparison of viral RNA copy number values at D1 showed a trend for higher replication of BA.1 compared to the D614G reference variant at 37°C (not significant; Fig. 1C) and confirmed that BA.1 replication was significantly higher than that of D614G at 33°C (Fig. 1D, *P* = 0.016). The replicative advantage of Omicron was apparent at early time points, but the replication of other VOCs caught up with that of BA.1 at the later day 4 (D4) time point, possibly due to a limitation in the target cell availability. An additional infection experiment performed on reconstructed nasal epithelia obtained from a single human donor confirmed the replicative advantage of Omicron BA.1 (Fig. S2), consistent with findings obtained on samples generated from pooled donors.

Computation of the ratio of viral RNA copies at 33°C over those at 37°C showed that viral replication tended to be slower at 33°C for all the viruses tested, with ratios below 1 in most cases, except for Omicron BA.1 at D4 (Fig. 1E; Fig. 1E). In addition, Omicron BA.1 was the only variant that showed a ratio significantly higher than that of the D614G reference strain at D2 (*P* < 0.001) and D4 (*P* < 0.01), emphasizing the efficient replication of the Omicron variant at a temperature more physiologically relevant for nasal infection.

We then performed infectivity measurements using the S-fuse assay, which quantifies SARS-CoV-2 infection by the emission of a GFP signal, after fusion of target U2OS-ACE2 cells expressing a GFP split system (9, 35, 36). Analysis of viral supernatants collected from two independent infection experiments showed that Omicron BA.1 had an early replicative advantage over D614G and Delta when propagated at 33°C (Fig. S3). Comparison of viral infectivities for infections carried out at 37°C showed a more variable advantage of Omicron, which proved detectable in one out of two experiments. Consistent with viral RNA release data, these experiments confirmed the replicative advantage of Omicron at the physiological temperature found in primary nasal epithelial cells.

### Omicron infection perturbs the epithelial barrier at physiological nasal temperature

**Fig 2:**
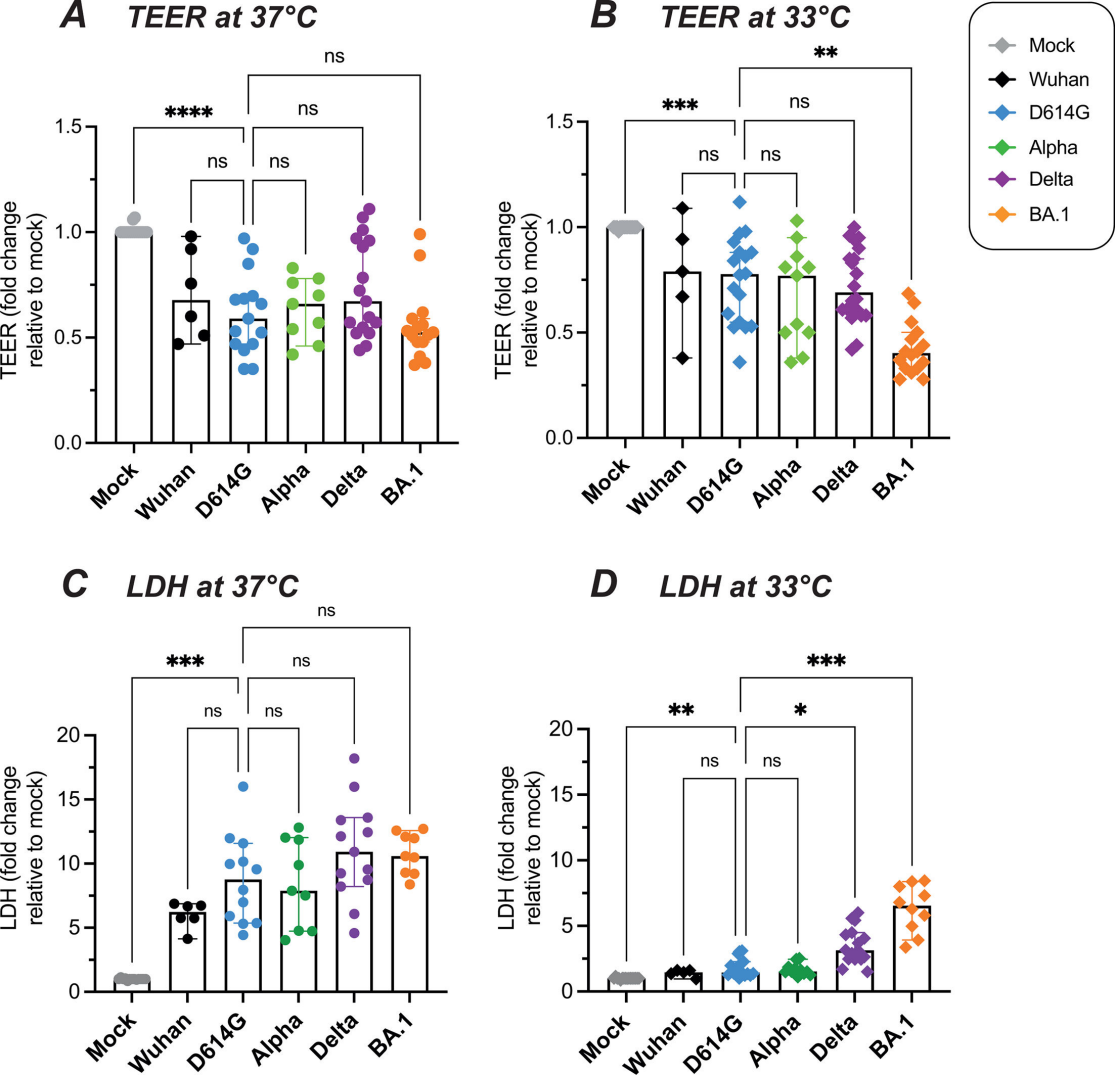
SARS-CoV-2 variants impair epithelial integrity. Infections with different SARS-CoV-2 variants were performed at an input equivalent to 10^8^ viral RNA copies. (**A, B**) Epithelial barrier function was assessed at 37°C (**A**) and 33°C (**B**) by measuring the TEER at 4 dpi. Relative TEER values reported to the Mock condition are shown. (**C, D**) The cytopathic effect was assessed at 37°C (**C**) and 33°C (**D**) by measuring the release of lactate dehydrogenase (LDH) in apical supernatants at 4 dpi. Relative LDH concentrations reported to the Mock condition are shown. (**A–D**) Medians and 95% confidence interval (CI) are reported. Statistical comparisons were done between the D614G reference and all other variants or the Mock condition, using the Kruskal-Wallis test with Dunn’s correction; ns: not significant; *P < 0.05; **P < 0.01; ***P < 0.001; ****P < 0.0001.

The trans-epithelial electrical resistance (TEER) measured between the upper and lower compartment of transwell cultures was used to evaluate the integrity of the epithelial barrier. All the variants tested induced a decrease in TEER at D4 as compared to mock-infected epithelia (Fig. 2A and B), indicating that SARS-CoV-2 perturbs epithelial barrier function. The extent of TEER decrease was comparable for all variants tested at 37°C, with a median decrease between 1.5- and 1.9-fold (Fig. 2A). In contrast, at the lower temperature of 33°C, the Omicron BA.1 variant caused a more marked TEER decrease than the reference D614G variant (median decrease of 2.5× vs 1.3×; *P* = 0.003; Fig. 2B). Thus, the Omicron variant proved particularly damaging for nasal epithelial integrity at the physiologically relevant temperature of 33°C.

We next asked whether epithelial damage was associated with cell death by measuring the apical release of LDH. An initial analysis of the kinetics of LDH detection showed that release of this enzyme was minimal at D2 and became clearly detectable at D4 (Fig. S4). Further measurements performed at D4 showed that all the variants tested induced an increase in LDH release (Fig. 2C and D), confirming the cytopathic effect of SARS-CoV-2 infection. The extent of LDH release did not differ significantly between the variants tested at 37°C (Fig. 2C). LDH release was overall lower at 33°C (Fig. 2D). However, at this lower temperature, the Delta and Omicron BA.1 variants showed a significantly increased cytopathic effect compared to the reference D614G variant (*P* = 0.04 for Delta; *P* = 0.0006 for BA.1). It was noteworthy that the Delta variant showed a clear cytopathic effect in the absence of a replicative advantage in epithelial cultures, a phenomenon that may be ascribed to the high fusogenicity of this variant (6, 37).

**Fig 3:**
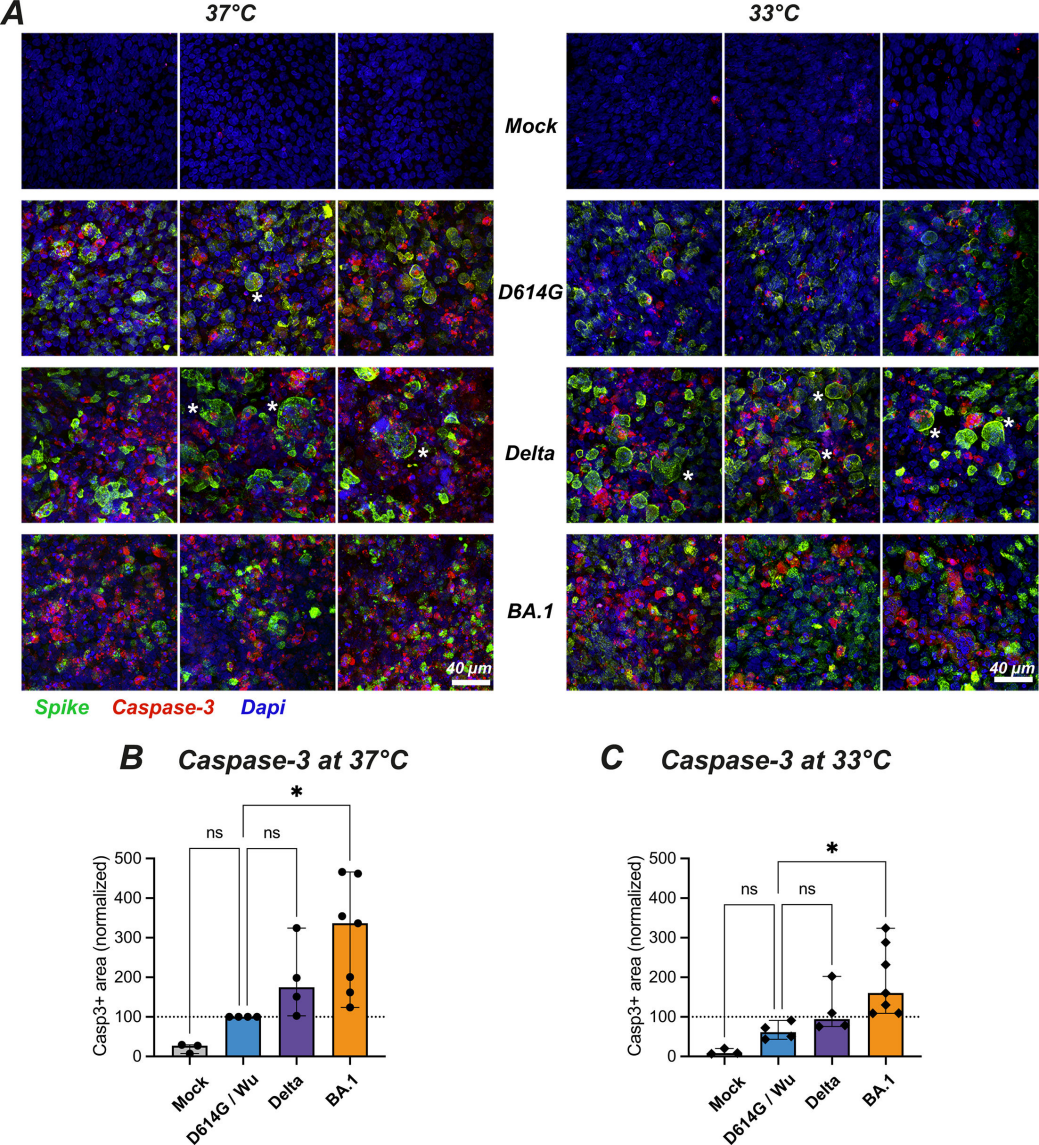
Temperature-dependent cytopathic effects induced by different SARS-CoV-2 variants. (**A**) Reconstructed nasal epithelia were Mock-infected (top line) or infected with different SARS-CoV-2 variants (D614G, Delta, and Omicron BA.1) at 37°C (left) or 33°C (right) and were fixed at 4 dpi and immunolabeled for cleaved caspase-3 (red), Spike (green) and nuclei (DAPI, blue). The scale bar represents 40 µm. The * symbol labels large syncytia. (**B–C**) Quantification of the cleaved caspase 3-positive area (casp3+) at 37°C (**B**) and 33°C (**C**), with values normalized to the D614G or Wuhan values (D614G/Wu) at 37°C. (**A–C**) Infections were performed at an input equivalent to 10^8^ viral RNA copies. Medians and 95%CI are reported. Statistical comparisons were done between the D614G/Wu reference and all other variants or the Mock condition, using the Kruskal-Wallis test with Dunn’s correction; ns: not significant; *P < 0.05.

To further investigate the cytopathic effect induced by the different SARS-CoV-2 variants, we evaluated by immunofluorescence the expression of the cleaved active form of caspase-3, an apoptosis effector protein. Cleaved caspase-3 was induced by infection at D4, and more so at 37°C than at 33°C (Fig. 3A). Fluorescence quantification showed that Omicron BA.1 was the most efficient at inducing cleaved caspase-3 at both temperatures (*P* < 0.05 in both cases), while Delta showed a nonsignificant trend for increase as compared to the D614G and Wuhan strains (Fig. 3B and C). The Spike and cleaved caspase-3 signals colocalized in some but not all infected cells, pointing to caspase-3 activation in infected cells but also possibly in bystander cells. It was interesting to note that large Spike+ cells corresponding to syncytia appeared more prominent for the Delta variant (Fig. 3A, third row), consistent with the highly fusogenic nature of this variant. Taken together, these findings documented a gradation in the caspase-3-mediated cytopathic effect, with maximal induction by Omicron BA.1, intermediate induction by Delta, and lower induction by other variants. Of note, Omicron significantly perturbed epithelial integrity and viability even at the physiological temperature of 33°C.

### Omicron infection induces the loss of motile cilia at physiological nasal temperature

**Fig 4:**
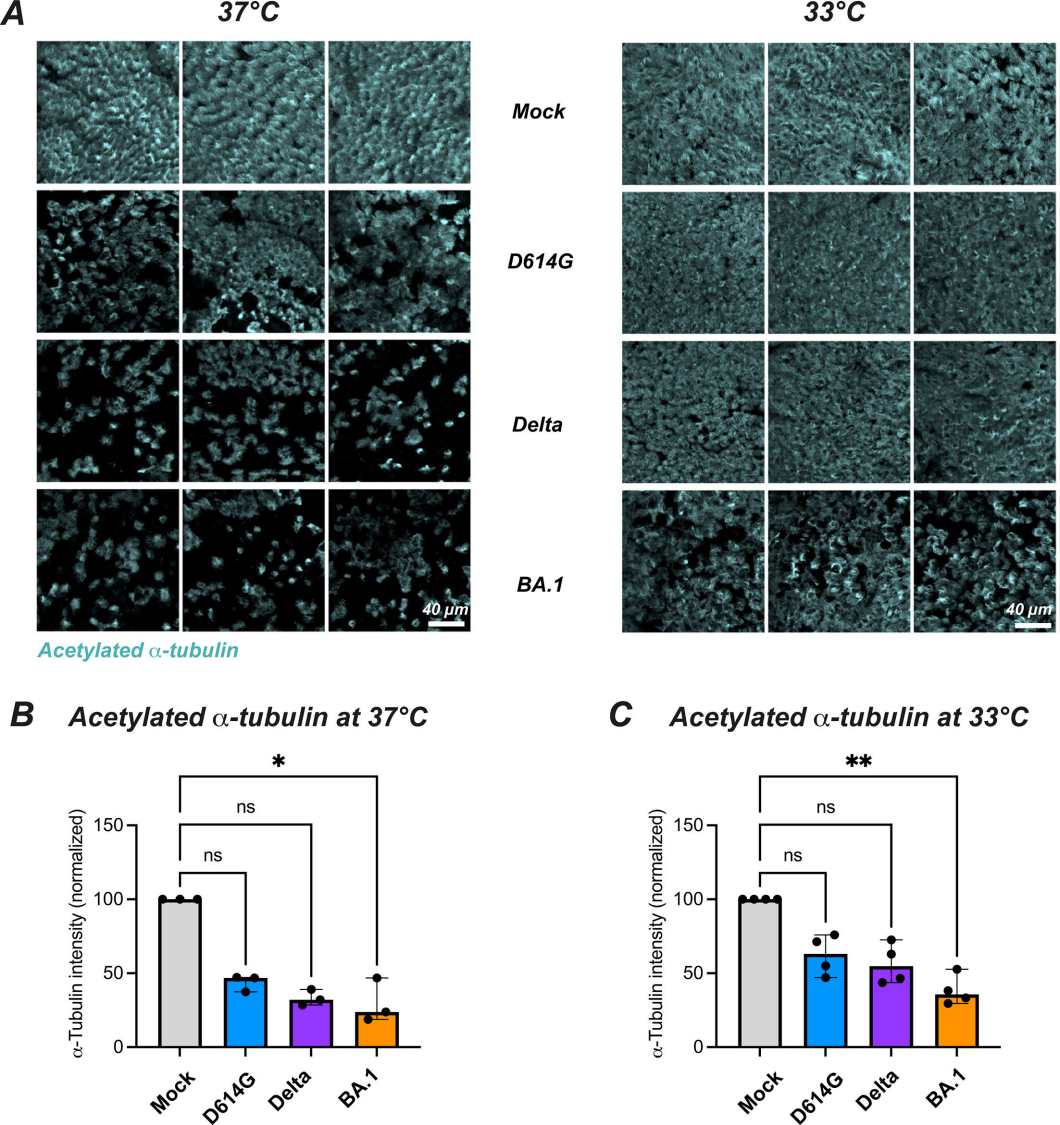
SARS-CoV-2 variants perturb the motile cilia layer in a temperature-dependent fashion. (**A**) Reconstructed nasal epithelia were mock-infected (top line) or infected with different SARS-CoV-2 variants (D614G, Delta, and Omicron BA.1) at an input equivalent to 10^8^ viral RNA copies. Cultures propagated at 37°C (left) or 33°C (right) were fixed at 4 dpi and immunolabeled for acetylated α-tubulin (cyan), which preferentially detects motile cilia. The scale bar represents 40 µm. (**B–C**) Quantification of the area positive for acetylated α-tubulin at 37°C (**B**) and 33°C (**C**), with values normalized to the Mock-infected condition. Medians and 95%CI are reported. Statistical comparisons were done between the Mock-infected sample and all the SARS-CoV-2 variant-infected samples, using the Kruskal-Wallis test with Dunn’s correction; ns: not significant; *P < 0.05; **P < 0.01.

We next evaluated the impact of the different viral variants on the motile cilia layer that covers airway epithelia. Reconstructed nasal epithelia collected at D4 post-infection were labeled for the acetylated form of α-tubulin, a tubulin isoform that is enriched in the axoneme of motile cilia. Immunofluorescence analysis showed that the three variants tested induced a loss of motile cilia at 37°C (Fig. 4A, left), with a decrease that was significant only for Omicron BA.1 (Fig. 4B, *P* = 0.026). The loss of cilia proved less marked at 33°C (Fig. 4A, right), with again a significant decrease seen only for BA.1 (Fig. 4C; *P* = 0.002).

**Fig 5:**
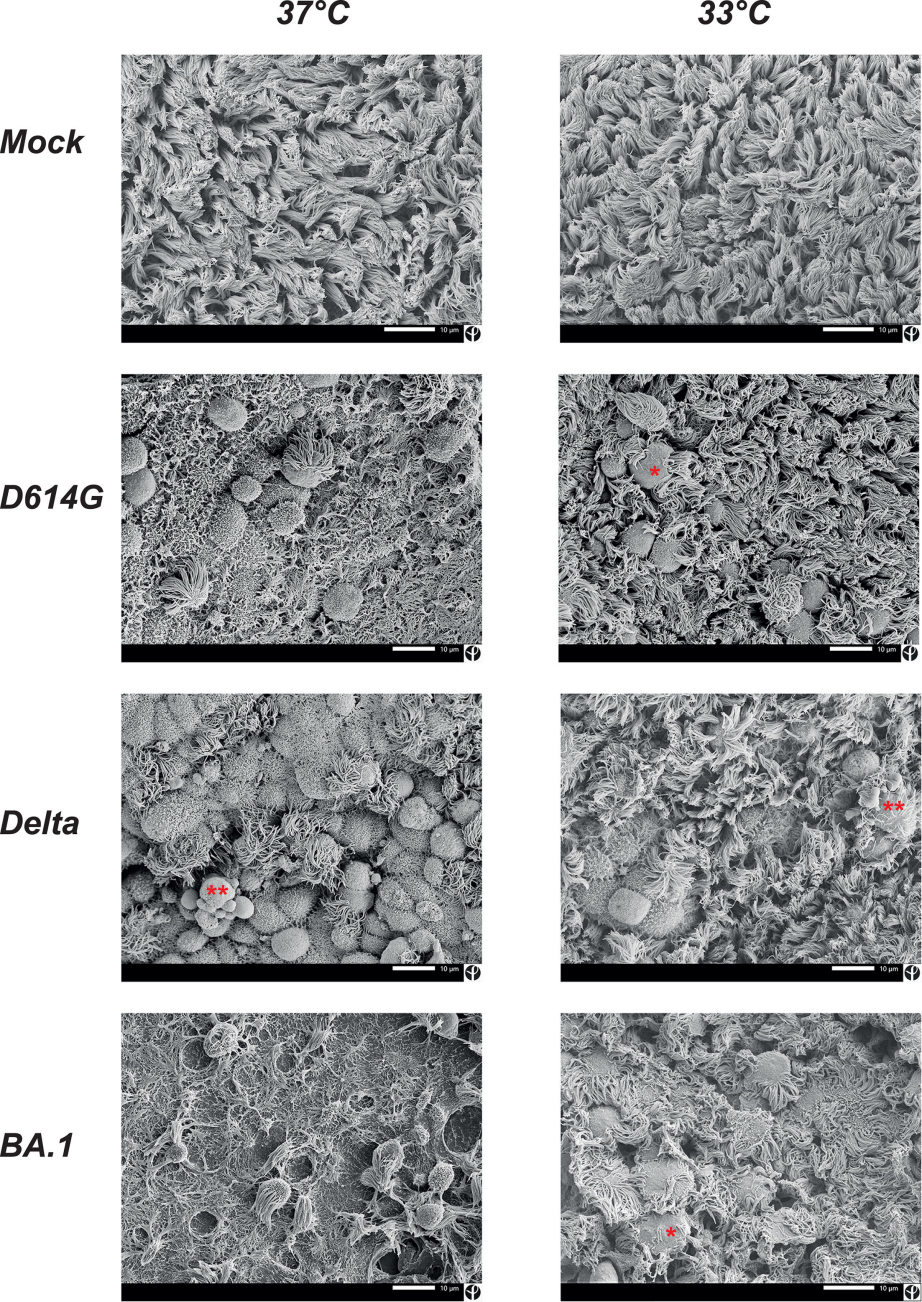
Infection by SARS-CoV-2 variants induces a loss of motile cilia. SEM images of reconstructed nasal epithelia at 4 dpi. The epithelia were infected at 37°C (left) or at 33°C (right) with the D614G, Delta, or Omicron, BA.1 variant (shown in rows 2, 3, and 4, respectively) or were Mock-infected (first row). The scale bar represents 10 µm. Red symbols: * membrane protrusion surrounding cilia; ** blebbing cell.

Examination of the infected epithelia by scanning electron microscopy (SEM) confirmed the disruption of the motile cilia layer by infection at D4, with the presence of multiple rounded cells with partial or complete deciliation, predominantly seen at 37°C but also detectable at 33°C (Fig. 5). The observation of membrane protrusions surrounding cilia suggested that engulfment may contribute to cilia loss. SEM analysis at higher magnification showed the accumulation of viral particles at the surface of partially deciliated cells (Fig. S5). Many of the rounded deciliated cells appeared pushed to the surface of the epithelial layer, consistent with the extrusion of dying epithelial cells. Rounded imprints corresponding to missing cells were prominent in BA.1-infected epithelia (Fig. 5, bottom left image). The detection of blebbing cells also pointed to the occurrence of apoptosis. Overall, infection by D614G, Delta, and BA.1 perturbed the ciliary layer at 37°C, while changes were more prominent for the BA.1 variant at 33°C.

### Omicron induces a broad transcriptional downregulation of ciliary genes but only a limited upregulation of host defense genes at physiological nasal temperature

To identify the transcriptional changes that may contribute to epithelial perturbation, we performed bulk RNA-seq analysis on epithelia at D2 post-infection. This early time point was chosen to better identify transcriptional patterns that may cause later functional and morphological changes.

**Fig 6:**
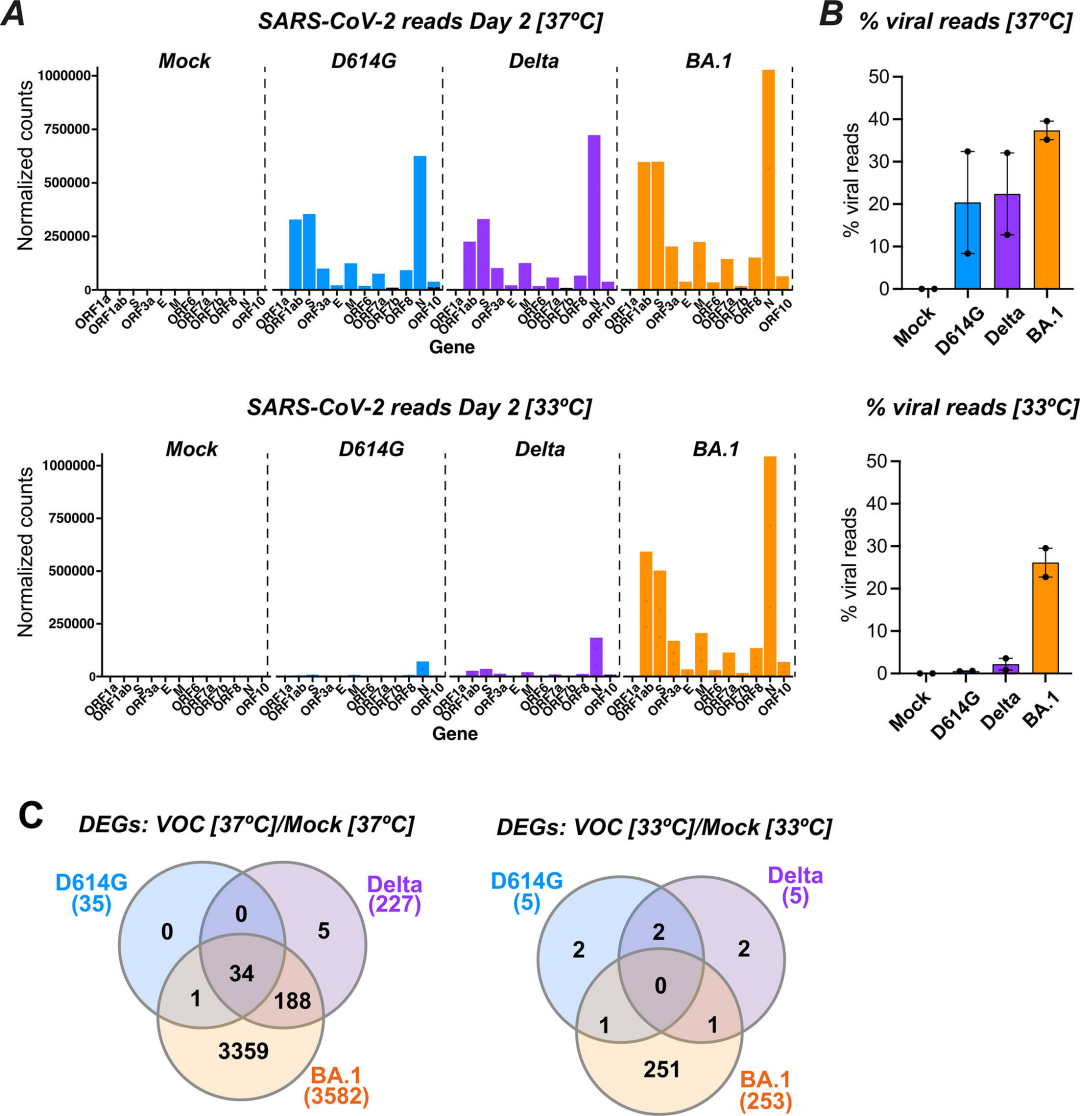
Temperature-dependent alteration of the cellular transcriptomic profile upon SARS-CoV-2 infection: A bulk RNA-seq analysis was performed at 2 dpi on reconstructed nasal epithelia either Mock-infected or infected with SARS-CoV-2 variants, using cultures grown at 37°C or 33°C. (**A**) Distribution of SARS-CoV-2 reads at 37°C (top graph) and 33°C (bottom graph), showing the mean normalized counts per million for the different SARS-CoV-2 transcripts (*n* = 2 replicates). (**B**) The percentage of viral reads among total mapped reads (cellular and viral) is reported for *n* = 2 replicate samples infected at 37°C (top) and 33°C (bottom). The median and 95%CI are reported. (**C**) Venn diagram showing the number of differentially expressed cellular genes (DEGs) shared between the different infected samples at 37°C (left graph) and 33°C (right graph). DEGs were determined with the DESeq2 package, with Mock-infected samples used as a reference. The total number of DEGs for each variant is reported below the variant name.

An initial analysis of viral gene reads showed that, at 37°C, viral expression was clearly detectable at D2 for the D614G, Delta, and Omicron BA.1 variants (Fig. 6A, top). The pattern of viral gene expression was comparable for the three variants, with N transcripts being the most abundant, followed by those coding for ORF1a and ORF1ab. The percentage of viral reads tended to be higher for BA.1 compared to D614G and Delta (Fig. 6B, top). It was notable that the percentage of viral reads reached above 30% of total mapped reads for epithelia infected by BA.1, highlighting how SARS-CoV-2 could usurp cellular resources for its own replication. The analysis of epithelia collected after 2 days of infection at 33°C showed a contrasting situation (Fig. 6A and B, bottom), with minimal detection of viral reads for the D614G and Delta variants, while the extent of BA.1 replication was high (26% of total mapped reads) and close to that observed at 37°C. The RNA-seq analysis thus confirmed the replicative advantage of the BA.1 variant at physiological nasal temperature.

DEGs were defined as cellular genes that showed at least a twofold change in expression upon infection with an adjusted *P* value < 0.05. The number of DEGs depended both on the nature of the infecting viral variant and on temperature (Fig. 6C). At 37°C, transcriptional dysregulation was low for D614G (*n* = 35 DEGs), moderate for Delta (*n* = 227), and marked for BA.1 (*n* = 3582). Thus, the impact of infection on cellular transcripts was strongly variant-dependent, while viral transcripts at the same time point differed only to a moderate extent between variants. The cumulative effects of slight differences in viral replication kinetics may contribute to this variable effect on cellular transcriptomes. At 33°C, the D614G and Delta variants induced only minimal changes in cellular gene expression (*n* = 5 DEGs for both viruses), while BA.1 caused clear cellular gene dysregulation (*n* = 253 DEGs), which remained however lower than that observed at higher temperature (Fig. 6C).

**Fig 7:**
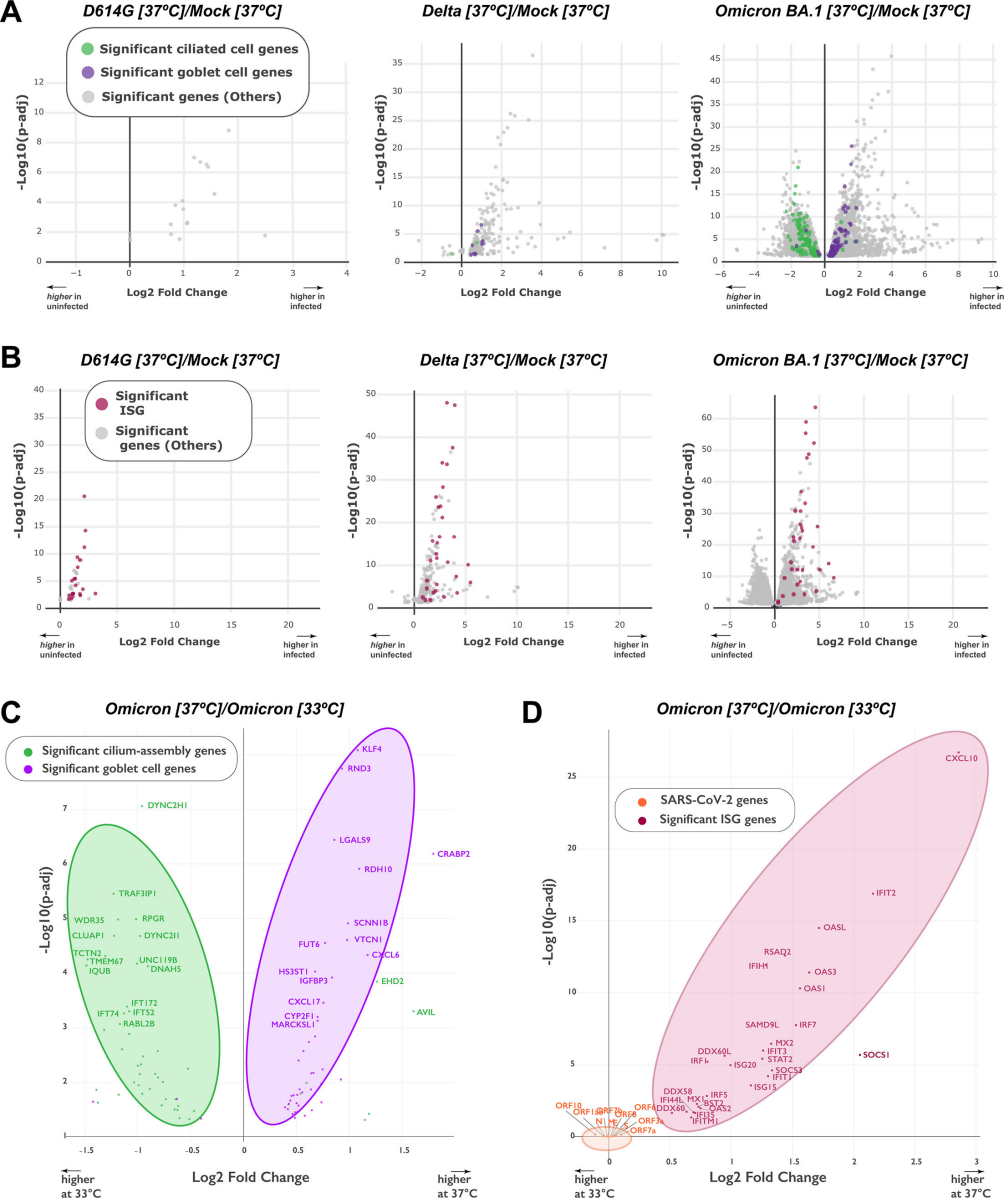
Transcriptional downregulation of ciliary genes and upregulation of IFN-stimulated genes (ISG) are variant- and temperature-dependent. (**A**) Volcano plots depicting the differential regulation of ciliated cell genes (green) and goblet cell genes (purple) at 37°C in variant-infected samples compared to Mock-infected samples at 2 dpi. Ciliated cell and goblet cell genes were defined by the clusterProfiler package. The log2 fold change in gene expression is shown on the X axis and the adjusted *P* value on the Y axis. Genes overexpressed in infected samples are distributed to the right on the X axis. Significant DEGs that do not belong to the ciliated cell or goblet cell category are represented in gray. (**B**) Volcano plots depicting the differential regulation of ISGs (red) and viral genes (orange) at 37°C in variant-infected samples compared to Mock-infected samples at 2 dpi. The representation is similar to that in (**A**). (**C, D**) Volcano plots showing the impact of temperature on the differential expressions of ciliated and goblet cell genes (**C**) or of ISGs and viral genes (**D**) in Omicron BA.1-infected samples at 2 dpi. Genes that are preferentially expressed at 37°C are distributed to the right on the X axis. Gene symbols are reported for the most dysregulated cellular genes and all viral genes.

Interestingly, functional enrichment analysis revealed that the biological processes dysregulated by infection at D2 depended on the viral variant considered (Fig. S6). At 37°C, the top two gene ontology (GO) terms associated with transcriptional changes were "defense response" to viruses and symbionts for D614G and Delta, while the top two GO terms were "cilium organization" and "cilium assembly" for BA.1. At the lower temperature of 33°C, the same cilium-related GO terms were the most dysregulated for BA.1, while changes in biological processes were minimal for D614G and Delta (counts ≤3). Based on these findings, we analyzed more in depth the transcriptional changes for genes involved in cilia structure and function (Fig. 7A). In a volcano plot analysis, we compared the expressions of genes specific for ciliated cells and goblet cells as a balance between these two cell types had been previously reported in airway epithelia (38). The analysis confirmed that Omicron BA.1 infection at 37°C induced a marked downregulation in the expressions of ciliated cell genes, with a concomitant upregulation in the expressions of goblet cell genes (Fig. 7A, right plot). In contrast, Delta infection caused only a slight upregulation of goblet cell gene expression, and D614G infection did not modulate ciliated nor goblet cell-specific genes at this early D2 time point (Fig. 7A, middle and left plots). The same analysis carried out on epithelial samples infected at 33°C for 2 days confirmed the decrease in ciliated cell gene expression and the increase in goblet cell gene expression induced by Omicron BA.1, while changes were not detected in these two gene sets upon D614G or Delta infection (Fig. S7A). Thus, Omicron BA.1 induced early D2 changes in transcriptional patterns that could account for the loss of motile cilia observed morphologically at the later D4 time point. The slower replication kinetics of D614G and Delta may explain the limited extent of transcriptional changes observed for these two variants at D2.

We then performed a volcano plot analysis for ISGs, which represent the major gene set modulated early during antiviral defense (Fig. 7B). Upon infection at 37°C, the three variants did induce ISG transcripts at D2, with a more marked upregulation for the BA.1 and Delta variants compared to D614G (Fig. 7B). In contrast, at 33°C, the induction of ISG transcripts remained minimal for Omicron and undetectable for the Delta and D614G variants (Fig. S7B). To further explore this temperature-dependent effect, we directly compared gene expression patterns induced by Omicron infection at 33°C and 37°C (Fig. 7C and D). These comparisons were not performed for D614G and Delta, which showed too few DEGs at 33°C. Omicron BA.1 was found to more efficiently downregulate ciliated cell genes and upregulate goblet cell genes at 37°C than 33°C (Fig. 7C), consistent with the trend for a higher motile cilia loss observed at 37°C by immunofluorescence (Fig. 4). The most significantly downregulated cilia genes observed at 37°C coded for *DYNC2H1*, a microtubule-associated dynein; *TRAF3IP1*, a microtubule-interacting protein associated with *TRAF3; RPGR*, a guanyl nucleotide exchange factor involved in ciliation; and *WDR*, a component of the intraflagellar transport complex. Thus, both structural and regulatory components of motile cilia were affected by Omicron infection.

Analyzing temperature effects on antiviral defense gene expression showed that Omicron BA.1 infection induced a markedly stronger upregulation of ISGs at 37°C than at 33°C (Fig. 7D). The most significantly upregulated ISG at 37°C included the chemokine *CXCL10*, which attracts immune cellular effectors; the *IFIT2* antiviral protein, which inhibits the expression of uncapped viral RNA; the *OASL* RNA-binding protein, which potentiates IFN production; and *RSAD2* or viperin, an enzyme that produces an antiviral ribonucleotide. Multiple mechanisms targeting RNA virus replication were thus induced by Omicron at 37°C. Viral transcripts were represented on the same volcano plot to evaluate the association between the triggering factors (viral RNA) and the epithelial antiviral response (ISG expression). Interestingly, while the difference in ISG expression was marked at 37°C vs 33°C, the difference in BA.1 viral expression was minimal. Indeed, viral transcripts were grouped close to the origin of the volcano plot, pointing to a lack of significant changes in viral expression when comparing the two temperatures (Fig. 7D, orange symbols). These findings highlight the capacity of Omicron BA.1 to replicate efficiently at physiological nasal temperature while inducing only a limited antiviral defense reaction in the epithelium.

### Limited induction of IFNs by Omicron at physiological nasal temperature

**Fig 8:**
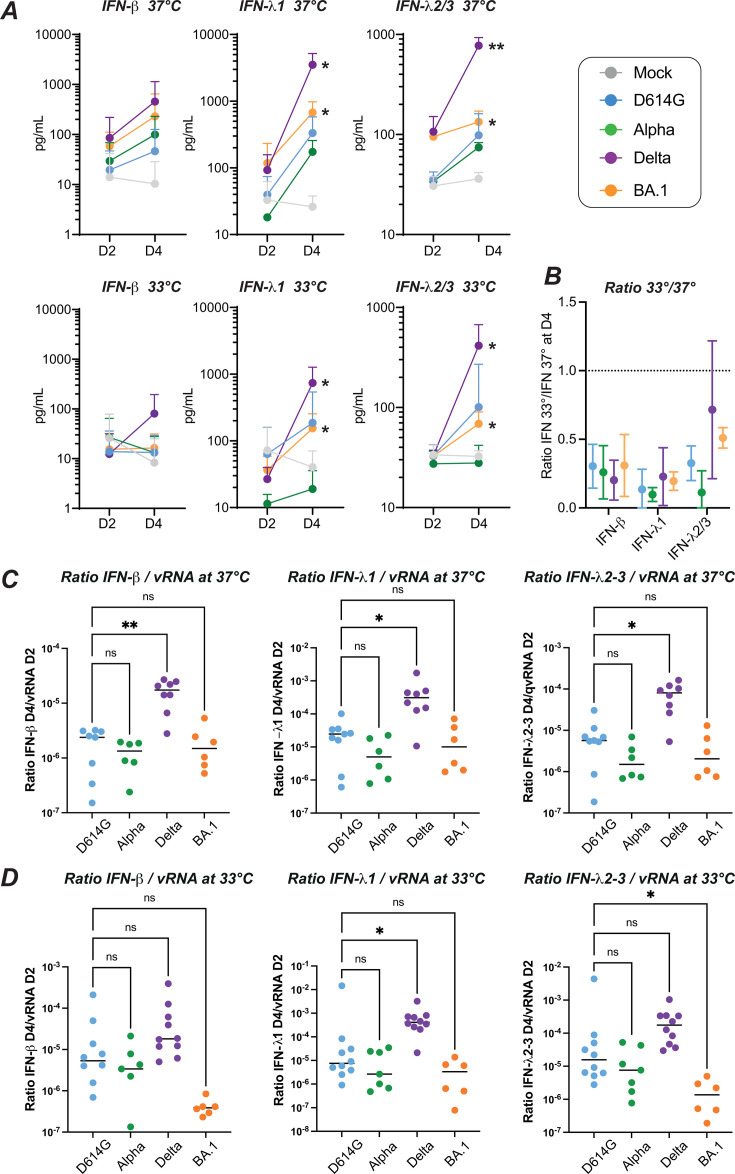
Differential induction of type I/III IFNs by SARS-CoV-2 variants (**A**) The induction of IFN-β (left), IFN-λ1 (middle), and IFN-λ2/3 (right) release in apical supernatants of reconstructed nasal epithelia infected at 37°C (top panels) and 33°C (bottom panels) was measured by digital ELISA (Simoa) and Legendplex assays. The mean ± SD are reported for *N* = 3 to 6 independent biological replicates. The induction of IFN between 2 dpi (D2) and 4 dpi (D4) was evaluated with a paired *t*-test. (**B**) The ratio of IFN measured at 33°C to that measured at 37°C is reported for the four variants tested (colored symbols) and the three IFNs measured (IFN-β, left; IFN-λ1, middle; IFN-λ2/3, right) at the D4 time point. The mean ± SD are reported for *N* = 4 to 5 independent biological replicates. The dashed line represents a ratio value of 1. (**C**) The ratio of IFN released at 4 dpi to the viral RNA released at 2 dpi is reported at 37°C (**C**) and 33°C (**D**) for IFN-β (left plots), IFN-λ1 (middle plots), and IFN-λ2/3 (right plots). Medians are reported. Statistical comparisons were done between the D614G reference variants and all the other variants tested, using the Kruskal-Wallis test with Dunn’s correction; ns: not significant; *P < 0.05; **P < 0.01.

To further evaluate the epithelial antiviral response upon SARS-CoV-2 infection, we directly measured the secretion of IFNs in apical culture supernatants at D2 and D4 (Fig. 8). Using high-sensitivity immunoassays (32, 39), we detected an induction of both type I (IFN-β) and type III (IFN-λ1 and IFN-λ2/3) IFNs between D2 and D4 at 37°C, with a positive trend for all the variants tested (Fig. 8A, top row). Of note, the Delta variant induced the strongest IFN induction at D4, while IFN concentrations observed for Omicron BA.1 appeared intermediate, and those observed for the other variants Alpha and D614G remained low (IFNλ1 induction between D2 and D4: *P*<0.05 for Delta and for BA.1. IFNλ2/3 induction between D2 and D4: *P* < 0.01 for Delta; *P* < 0.05 for BA.1). A similar phenomenon was observed in epithelial cultures infected at 33°C (Fig. 8A, bottom row), with a more efficient induction of type I and type III IFNs by Delta compared to the other variants tested, including Omicron. Indeed, Delta was the only variant to induce a detectable production of IFN-β at physiological nasal temperature.

To compare the production of IFNs at the two temperatures tested, we computed the ratio of concentrations measured at 33°C to those measured at 37°C for the D4 timepoint (Fig. 8B). The production of IFNs proved consistently weaker at 33°C, as indicated by ratios below 1 for all the IFNs tested. For type III IFNs, the ratios tended to be higher for Delta and Omicron compared to Alpha. However, taken together, all the SARS-CoV-2 variants tested triggered lower antiviral responses at physiological nasal temperature.

An intervariant comparison of apical IFN concentrations measured at D4 at 37°C (Fig. S8A) confirmed that Delta was the only variant to induce a significantly higher IFN secretion compared to the reference variant D614G, independently of the IFN tested (*P* < 0.01 for IFN-β, IFN-λ1, and IFN-λ2/3). Omicron induced an intermediate IFN secretion that did not differ significantly from that measured for D614G. A similar pattern was observed at 33°C (Fig. S8B). To determine whether these findings applied to other IFNs, we measured the secretion of IFN-α at D4 in a subset of experiments (Fig. S8C). We observed a trend for a higher induction of IFN-α by Delta compared to Omicron at 37°C, consistent with findings obtained for other type I and type III IFNs. In contrast, no induction of IFN-α could be detected at 33°C for all the variants tested, suggesting that IFN-α induction was highly temperature-dependent.

It was intriguing that Omicron BA.1 induced only a moderate induction of IFNs at 33°C, while this variant had an early replicative advantage at this temperature. To further explore this point, we computed the ratio of IFN secreted at D4 reported to the number of inducing viral RNA copies measured at the early D2 time point. At 37°C, the Delta variant showed significantly higher IFN to viral RNA ratios compared to the D614G reference, while Omicron BA.1 showed ratios equivalent to those of D614G (Fig. 8C). Interestingly, at 33°C, Omicron BA.1 showed lower IFN to viral RNA ratios compared to D614G (*P* < 0.05 for IFNλ2/3), while a trend for higher ratios was maintained for Delta (*P* < 0.05 for IFNλ1). This analysis confirmed that Delta was a potent inducer of type I and type III IFNs. In contrast, Omicron BA.1 was characterized by a weak induction of IFNs relative to its replication capacity, especially at physiological nasal temperature.

## DISCUSSION

This study highlights that the SARS-CoV-2 Omicron variant evolved several traits that converged in facilitating viral spread. First, Omicron displayed an early replicative advantage in primary human nasal epithelial cells, which represent the main source of transmissible virus. Second, the increase in Omicron replication was more notable at 33°C, the temperature typically found in human nasal passages, resulting in a physiologically relevant replicative advantage. Third, while Omicron did not entirely disrupt the epithelial structure when replicating at 33°C, it did cause measurable decreases in epithelial integrity and in motile cilia coverage. These changes are likely to perturb the mechanism of mucociliary clearance, as we reported previously (32), and could thus facilitate the spread of viral particles within the airways. Fourth, at this physiological temperature, Omicron remained a moderate inducer of IFN responses relative to its replicative capacity in the nasal epithelium. Considering the key role of the type III IFN response in limiting the early spread of viruses at epithelial surfaces (40, 41), it is relevant that Omicron induced comparatively less IFN-λ1 and IFN-λ2/3 than the Delta variant. The combined advantages of increased viral replication, perturbation of motile cilia, and escape from the innate antiviral response in the nasal epithelium likely contributed to the worldwide spread of the original Omicron BA.1 variant and to the continued dominance of Omicron-derived variants since 2022. While escape from the adaptive immune response clearly played an important role in Omicron emergence (8, 9), our findings argue for an additional role of intrinsic viral properties in the evolutionary success of the Omicron variant.

The IFN response is a key determinant of COVID severity, as shown by the association of inborn errors in the type I IFN pathway and critical COVID pneumonia (42). The importance of the IFN pathway is also supported by the detection of auto-antibodies capable of neutralizing type I IFNs in up to 20% of patients who had fatal COVID (43). A decreased IFN response could be directly observed in the nasopharyngeal swabs from patients with severe COVID (38). In the face of the major selective pressure exerted by the IFN response, SARS-CoV-2 has evolved a variety of countermeasures, with multiple viral genes involved in dampening the induction of IFNs and/or inhibiting the function of ISGs (44, 45). Indeed, SARS-CoV-2 was shown to be more efficient at escaping the innate antiviral response than common cold coronaviruses, helping explain why the former is more pathogenic (46).

Of interest, successive SARS-CoV-2 variants have shown an evolution in their capacity to escape the innate antiviral response. The early variant Alpha was shown to induce lower levels of IFNs than the original SARS-CoV-2 strain, due to increased expression of viral genes involved in innate immune antagonism, such as ORF6, ORF9b, and a truncated form of the nucleocapsid (47, 48). Studies of later variants have yielded variable findings, with some studies showing an increasing capacity to escape the antiviral effects of type I IFNs from Alpha to Delta to Omicron (30), others showing a better escape for Alpha and Omicron compared to Delta (31, 49), and still others reporting a more efficient escape for Delta compared to Omicron BA.1 (50). One reason for these divergent findings could be that some studies were performed on transformed cell lines such as Calu-3, in which Omicron shows an attenuated phenotype, in contrast to the fast-replicating phenotype observed in primary epithelial cells. Another confounding factor may be the procedure used to titrate viral stocks, which often relies on serial dilutions in transformed cell lines such as Vero-E6-derived cells. As Omicron shows an attenuated phenotype in such cell lines, the resulting viral stocks tend to be underestimated in terms of infectivity for primary cells. To avoid these caveats, we chose to normalize the viral stocks based on viral RNA content, which is a trigger of the IFN response (51). Further, we chose to analyze viral replication kinetics and IFN induction in a human primary nasal cell-based model, which is more likely to yield information relevant to human physiology.

Based on this strategy, we observed that the induction of type I and type III IFNs in primary nasal epithelial cells was low for Alpha, intermediate for Omicron, and significantly higher for Delta. To consider the faster replication kinetics of Omicron, and hence the more rapid accumulation of viral RNA acting as a pathogen-associated molecular pattern (PAMP), we computed the ratio of produced IFN to early viral RNA copies. This analysis pointed to a relatively low intensity of the IFN response to Alpha and Omicron, compared to a significantly higher induction of type I and III IFN for Delta. Omicron may be qualified as a "stealthy" variant as it combines a high replicative capacity with a limited triggering of the IFN response at physiological nasal temperature. This does not imply an entire lack of epithelial antiviral response as a subset of ISGs were still induced by Omicron at 33°C but points to a dampening of the antiviral response as compared to previous SARS-CoV-2 variants. A possible reason for the strong innate response to Delta may be the highly fusogenic phenotype of this variant (6, 37) as fusion is *per se* a triggering event for the IFN response (52). Fused syncytia are thought to have short life spans and to release damage-associated molecular patterns (DAMPs) when dying. It is thus relevant that we observed the formation of larger syncytia in the Delta-infected cultures, consistent with a highly fusogenic phenotype for this variant. A limitation of our study is that the reconstructed epithelium model used is devoid of immune cells, so we cannot evaluate the contribution of resident and inflammatory leukocytes to the mucosal IFN response. It is interesting, however, that an *in vivo* study of the innate response in nasopharyngeal swabs obtained during acute COVID found an inverse correlation between nasal ISG expression and COVID severity in patients infected with the ancestral and Delta variants, but not with the Omicron variant (53). These *in vivo* findings are compatible with a stronger impact of the IFN response on Delta than on Omicron infections, consistent with our findings in primary nasal epithelial cells. Also relevant is a recent study showing that later Omicron variants, including BA.4 and BA.5, have further increased their suppression of innate immunity compared to BA.1 and BA.2, pointing to a continued evolution of SARS-CoV-2 variants toward improved innate immune evasion (29).

All the variants tested could replicate at both 33°C and 37°C, consistent with the dual tropism of SARS-CoV-2 for the upper and lower respiratory tract (54–56). This contrasts with the preferential replication of common cold coronaviruses at 33°C, in line with their restriction to the upper respiratory tract (57). At the early D2 time point, the Omicron variant had already reached its replication plateau when grown at 33°C, while the induction of ISGs proved minimal or undetectable at this temperature. Furthermore, at D2, the production of type I and type III IFNs was not induced in Omicron-infected cultures grown at 33°C. These observations fit with the long-held notion that the IFN response is inefficient and delayed at lower temperature, mostly due to limited transcription of ISGs and IFN genes (58, 59). Our data quantifying IFN-β and IFN-λ suggest that this temperature effect may also impact IFN protein secretion. It was interesting that Omicron showed an early replicative advantage over other SARS-CoV-2 variants, particularly in the low-temperature condition. This observation suggests that Omicron is not only efficient at evading the IFN response, but that it also possesses other fitness-enhancing properties independently of its innate response evasion capacity.

Structural features may contribute to Omicron’s fitness at lower temperature as its Spike protein was shown to have better thermostability, with a tighter packing of the three receptor-binding domains in their down state (60, 61). The viral attachment step is also likely involved as Omicron was proposed to have a higher affinity for its receptor ACE-2 (23, 24) and a higher capacity to bind the plasma membrane of its target cells (25, 26). The SARS-CoV-2 fusion step depends on lipid membrane reorganization and is known to be temperature-dependent, with a lower efficiency at 33°C than at 37°C (62). As the Omicron Spike was proposed by some authors to switch more easily to a fusogenic conformation, it is possible that Omicron better overcomes the temperature-dependent barrier to fusion (25). On the other hand, a more rapid switch to a fusogenic conformation may lead to viral inactivation by fusion with extracellular vesicles, which can act as traps for incoming virions (63). It is thus interesting that the release of extracellular vesicles is decreased at lower temperature, which may facilitate the spread of fusion-prone variants at 33°C. The nature of the Omicron entry pathway remains to be precisely defined as several groups have reported increased Omicron entry through a cathepsin-dependent endosomal pathway (27, 28), while others have highlighted an increased dependence of Omicron entry on metalloproteases (15, 64), and still others report a continued dependence on the surface protease TMPRSS2 to trigger Omicron Spike fusion (65). Further studies are thus warranted to precisely characterize the post-attachment steps involved in Omicron entry in primary cells and determine whether they contribute to the replicative advantage of this variant at physiological nasal temperature.

Of note, Omicron perturbed the integrity of the motile cilia layer more efficiently than other variants. The differences were marked at lower temperature, with a significant decrease in TEER values compared to the reference D614G variant, indicative of increased epithelial permeability. In addition, Omicron induced increased cell death and cilia loss at 33°C compared to other variants. Motile cilia loss can have several consequences on viral dissemination. At the cellular level, it can facilitate the dissemination of viral particles, which are released at the plasma membrane and will not have to cross a layer of packed cilia to reach neighboring cells and/or the mucus layer. The loss of motile cilia may also facilitate the passage of actin protrusions that push recently released SARS-CoV-2 virions toward the mucus layer (26). At the epithelium level, the loss of motile cilia inhibits mucociliary clearance as viral particles become trapped in a mucus layer that is not propelled anymore by ciliary beating (32). This may locally limit the spread of viral particles to neighboring ciliated cells as viral particles become less motile (66). On the other hand, at the organ level, the loss of mucociliary clearance prevents the removal of viral particles trapped in mucus and their redirection toward the pharynx (67, 68). The loss of this clearance mechanism likely facilitates the viral invasion of epithelia situated lower in the respiratory tract (69) and thus promotes viral dissemination within the infected organism. Omicron outbreaks in Asia have been associated with a high rate of bacterial and fungal superinfections, which may also reflect an impairment of mucociliary clearance (70, 71). It is interesting that the Omicron variant can perturb mucociliary clearance at the temperature found in nasal passages, suggesting that this property is physiologically relevant and beneficial to the evolutionary fitness of this variant.

In conclusion, the present study highlights multiple features that likely contributed to the efficient transmission and worldwide dissemination of Omicron. This SARS-CoV-2 variant appears particularly adapted to the upper respiratory tract based on its early replicative advantage at the temperature found in nasal passages, its capacity to perturb the motile cilia layer, and its limited induction of the IFN epithelial response. Further development of 3D-tissue models that include innate and adaptive immune cells should help achieve an integrated view of the parameters that control SARS-CoV-2 variant dissemination in the upper airways.

## MATERIALS AND METHODS

### SARS-CoV-2 infection of reconstructed human nasal epithelia

Reconstructed human nasal epithelial cultures (MucilAir) differentiated *in vitro* for at least 4 weeks were purchased from Epithelix (Saint Julien-en-Genevois, France). To minimize individual variability, each reconstructed epithelium was derived from nasal cells obtained from a pool of 14 donors. Epithelia generated from three distinct pools of donors were used in the present study. Information on the composition of these three pools (age and sex of donors) is provided in Table S1. Cultures were maintained in ALI conditions in transwells, with 700 μL of MucilAir medium (Epithelix) in the basal compartment and kept at 37°C or 33°C under a 5% CO_2_ atmosphere. For SARS-CoV-2 infection, the apical side of ALI cultures was washed 20 min at 37°C or 33°C in Mucilair medium to remove mucus. Cells were then incubated with the equivalent of 10^6^ RNA viral copies/µL of viral stock for each of the SARS-CoV-2 variants tested: Wuhan, D614G, Alpha, Beta, Gamma, Delta, and Omicron BA.1. The source of the viral isolates used is reported in Table S2. The viral input was diluted in DMEM to a final volume of 100 μL and left on the apical side for 4 h at 37°C or 33°C. Control wells were mock-treated with DMEM (Gibco) for the same duration. Viral inputs were removed by washing three times with 200 μL of phosphate-buffered saline (PBS) (5 min at 37°C or 33°C) and once with 200 μL MucilAir medium (20 min at 37°C or 33°C). The basal medium was replaced every 2–3 days. Apically released viruses were collected by incubation with 200 μL MucilAir medium (20 min at 37°C or 33°C) at 1, 2, and 4 dpi. The integrity of the epithelia was monitored by measuring the TEER at the same days. To do so, after apical wash, the transwells were transferred into a new 24-well plate and DMEM was added to both the apical (200 μL) and basal (700 μL) sides. The TEER was then measured using an Evom3 ohmmeter (World Precision Instruments). At day 4, cultures were fixed in paraformaldehyde 4% for 30 min, washed twice with PBS, and stored in PBS at 4°C until immunofluorescence staining.

### Viral RNA quantification

Culture apical supernatants collected at 1, 2, and 4 dpi were inactivated for 20 min at 80°C and then subjected to RT-qPCR analysis, using a Luna Universal One-Step RT-qPCR Kit (New England Biolabs), following the manufacturer’s instructions. SARS-CoV-2 RNA was quantified in a final volume of 5 μL per reaction in 384-well plates using SARS-CoV-2 N-specific primers (Forward 5′-CGA AGG TGT GAC TTC CAT G-3′; Reverse 5′- TAA TCA GAC AAG GAA CTG ATT A-3′) on a QuantStudio 6 Flex thermocycler (Applied Biosystems). A standard curve was performed in parallel using purified SARS-CoV-2 viral RNA (EURM019, Sigma Aldrich).

### S-Fuse infectivity assay

Infectious titers were quantified by automated imaging of cell-cell fusion in a GFP-split cell system. U2OS-ACE2 GFP1–10 and U2OS-ACE2-GFP11 cells, also termed S-Fuse cells, become GFP+ when they fuse together upon productive infection by SARS-CoV-2 (36). The S-Fuse cells tested negative for mycoplasma. S-Fuse cells were mixed (at a 1:1 GFP1−10/GFP11 ratio) and plated at 8 × 10^3^ cells per well in a μClear 96-well plate (Greiner Bio-One). The tested SARS-CoV-2 infection supernatants were serially diluted and added to S-Fuse cells. Post 18 h, cells were fixed with 2% paraformaldehyde (electron microscopy # 15714-S), washed in PBS, and stained with Hoechst at a 1:1,000 dilution (Invitrogen, H3570). Images were acquired automatically using an Opera Phenix high-content confocal microscope (PerkinElmer). The GFP+ area, the number of GFP+ objects, and the number of nuclei per well were quantified using the Harmony software (PerkinElmer). The S-fuse assay has been previously validated for the comparison of SARS-CoV-2 variant infectivities and antibody neutralization capacities (9, 35).

### Immunofluorescence labeling

Membranes supporting the fixed MucilAir cultures were cut in four using a scalpel blade. Membrane pieces were placed in 10 μL drops of PBS onto parafilm and then permeabilized in PBS 0.5% Triton-X100 for 20 min at room temperature (RT). Samples were blocked in PBS with 0.1% Tween, 1% bovine serum albumin (BSA), 10% fetal bovine serum, and 0.3 M glycine for 30 min at RT. Samples were then incubated overnight at 4°C with the following antibodies: pan-SARS-CoV-2 anti-Spike mAb102 human monoclonal antibody (72) at 1 μg/mL; rabbit anti-cleaved caspase 3 primary antibody (9661, Cell Signaling; 1:100 dilution); AF647-conjugated rabbit anti-alpha acetylated tubulin (ab218591, Abcam; 1:100 dilution). After washing, a secondary anti-human IgG antibody conjugated to AF555 (A-21422, Thermo Fisher Scientific) was added at 1:400 for 1 h at RT. Primary and secondary antibodies were diluted in PBS with 0.1% Tween and 1% BSA. Samples were counterstained with Hoechst and mounted in FluoromountG (Thermo Fisher Scientific) before observation with an LSM 710 confocal microscope (Zeiss) or with a BC43 spinning disk confocal microscope (Andor).

### LDH cytotoxicity assay

Diluted apical culture supernatants (1:25) were pretreated with Triton-X100 1% for 2 h at RT for viral inactivation. LDH dosage was performed using the LDH-Glo Cytotoxicity Assay kit (Promega) following the manufacturer’s instructions. Luminescence was measured using an EnSpire luminometer (PerkinElmer).

### Cytokine measurements

Apical culture supernatants were pretreated with Triton-X100 1% (final concentration) for 2 h at RT for viral inactivation. IFN-β protein was quantified using Simoa digital ELISA technology as previously described (32, 39). Alternatively, IFN-β and IFN-α were quantified simultaneously in a Simoa multiplexed assay. Briefly, the inactivated supernatant was diluted 1:10 in Diluent B (Quanterix, Billerica, MA, USA) containing 1% Triton-X100 (final concentration). IFN-α and IFN-β concentrations were quantified with a Simoa assay developed on an HD-X instrument with Quanterix Homebrew kits. The assay configuration was a 2-step triplex associating IFN-α, IFN-β, and IFN-λ3 (data not shown), using 150 pM SBG and 50 uL RGP reagents. The IFN-α plex quantifies all IFN-α subtypes with a similar sensitivity, as previously reported (39), with results expressed in IFN-α17 equivalents. The antibodies used were cloned from APECED/APS1 patients. The 8H1 antibody clone was used as a capture antibody after coating on paramagnetic beads (0.3 mg/mL); the 12H5 clone was biotinylated (biotin/antibody ratio = 30:1) and used as the detector at a concentration of 0.3 µg/mL. The recombinant human IFN-α17 protein (#11150, PBL Assay Science, Piscataway, NJ, USA) was used as a reference to quantify concentrations. The limit of detection was between 0.59 and 1.43 fg/mL equivalent IFN-α17, including the dilution factor. The IFN-β plex assay is described more in detail in 73. Briefly, the 710906-9 IgG1-κ mouse monoclonal antibody (PBL Assay Science) was used as a capture antibody to coat paramagnetic beads (0.3 mg/mL); the 710323-9 IgG1-κ mouse monoclonal antibody (PBL Assay Science) was biotinylated (biotin/antibody ratio = 40/1) and used as the detector antibody at a concentration of 0.3 µg/mL. The recombinant IFN-β protein (#11415, PBL Assay Science) was used as reference to quantify concentrations. The limit of detection was between 0.01 and 0.03 pg/mL IFN-β, including the dilution factor.

#### Quantification of IFN-λ1 and IFN-λ2/3 proteins using Legendplex technology

On the day of assaying, supernatants were centrifuged at 300 *g* for 5 min. The concentration of 13 different analytes was measured using the LEGENDplex Human Anti-Virus Response Panel (13-plex) with a V-bottom plate kit (cat. No. 740390), and the manufacturer’s protocol was followed (BioLegend, San Diego, California, USA). Plates were read on a BD Fortessa II 5-color flow cytometer (Franklin Lakes, New Jersey, USA), and the resulting mean fluorescence intensity data were analyzed using the BioLegend portal to predict the analyte concentration (https://legendplex.qognit.com/).

### Scanning electron microscopy

PFA-fixed epithelial samples were post-fixed with 2.5% glutaraldehyde in 0.1 M cacodylate buffer for 1 h at RT. Samples were then washed in 0.1 M cacodylate buffer and several times in water and then incubated in 1% osmium tetroxide for 1 h. After dehydration by incubation in increasing concentrations of ethanol (35%, 70%, 85%, 95%, and 100%), samples were treated with hexamethyldisilazane for 10 min for chemical critical point drying. Specimens were then sputter-coated with a gold/palladium conductive layer up to 9 Å using a gun ionic evaporator PEC 682. Images were acquired on a JEOL JSM 6700F field emission SEM operated at 7 kV.

### RNA-seq

#### Sample preparation and sequencing

After 2 days of infection, the epithelial cultures were washed in cold PBS and then lysed in 150 μL of TRIzol reagent (Thermo Fisher Scientific) added to the apical side of the insert. RNAs were purified using the Direct-zol miniprep kit (ZR2080, Zymo Research). RNA integrity and concentration were verified by Bioanalyzer (Agilent Technologies). mRNA enrichment, strand-specific library preparation, and paired-end sequencing (100 bp) on the DNBSEQ platform were performed at BGI (Poland).

#### Processing of raw sequencing data

Raw paired-end RNA-sequencing data obtained from BGI were analyzed using FastQC v0.11.9 for quality control (74) and trimmed with fastp v0.23.2 (75). Trimmed reads were mapped using HISAT2 v2.2.1 (76) to the following reference genomes: concatenated GRCh38 (human) genome and wuhCor1 (SARS-CoV-2) reference genomes obtained from the UCSC Genome Browser (http://hgdownload.soe.ucsc.edu/goldenPath). Duplicate alignments were labeled with the markDuplicates function from Picard v2.23.4 (http://broadinstitute.github.io/picard/). Marked alignment files were further processed using Sambamba 0.7.1 (77) keeping only uniquely mapping reads after duplicate removal.

#### Analysis of RNA-seq data

Mapped reads were quantified with featureCounts v2.0.0 using GRCh38 Gencode v36 annotation, following differential expression analysis with DESeq2 (78) with two replicates per condition. Genes were considered differentially expressed with an absolute log_2_ fold change value > 1 and adjusted *P*-value < 0.05. Functional enrichment analysis was performed using the clusterProfiler (79) and enrichR packages (80). Data visualization, including volcano plots, was carried out using custom scripts with the ggplot2 R package (81). To quantify the proportion of viral reads within each sample, the number of all mapped reads aligning to the SARS-CoV-2 genome was calculated using featureCounts v2.0.0 and further normalized by the total number of mapped reads per sample.

### Statistics and reproducibility

Statistical analyses were performed with the Prism software v8.4.3 (GraphPad) for all figures except for RNA-seq analyses described above. The nonparametric Kruskal-Wallis test with Dunn’s correction for multiple testing was used in all cases, except for paired analyses, where a paired *t*-test was used. All tests were two-sided. Statistical significance was assigned when *P* values were <0.05. The nature of statistical tests and the number of experiments (n) are reported in the figure legends.

## Data Availability

The data underlying this article are available in NCBI Gene Expression Omnibus (GEO) at https://www.ncbi.nlm.nih.gov/geo/ and can be accessed under accession no. GSE295759.

## SUPPLEMENTAL MATERIAL

Fig. S1 jvi.02008-25-s0001.pdf Viral replication of additional SARS-CoV-2 variants.

Fig. S2 jvi.02008-25-s0002.pdf Infections on nasal epithelia derived from a single donor.

Fig. S3 jvi.02008-25-s0003.pdf Viral infectivity analysis.

Fig. S4 jvi.02008-25-s0004.pdf Kinetics of LDH release.

Fig. S5 jvi.02008-25-s0005.pdf Scanning electron micrographs of deciliated cells.

Fig. S6 jvi.02008-25-s0006.pdf Gene enrichment analysis.

Fig. S7 jvi.02008-25-s0007.pdf Volcano plot analysis.

Fig. S8 jvi.02008-25-s0008.pdf Interferon production at day 4.

Table S1 jvi.02008-25-s0009.pdf Information on epithelial cell donors.

Table S2 jvi.02008-25-s0010.pdf Viral isolates used in the study.

ASM does not own the copyrights to Supplemental Material that may be linked to, or accessed through, an article. The authors have granted ASM a non-exclusive, world-wide license to publish the Supplemental Material files. Please contact the corresponding author directly for reuse.
