# Supplementary material for: Stealth replication of SARS-CoV-2 Omicron in the nasal epithelium at physiological temperature
Source: J Virol. 2025 Dec 19;100(1):e02008-25. doi: 10.1128/jvi.02008-25 (PMC12817898; doi:10.1128/jvi.02008-25)
Supplement: Fig. S1 — Viral replication of additional SARS-CoV-2 variants. [file jvi.02008-25-s0001.pdf]

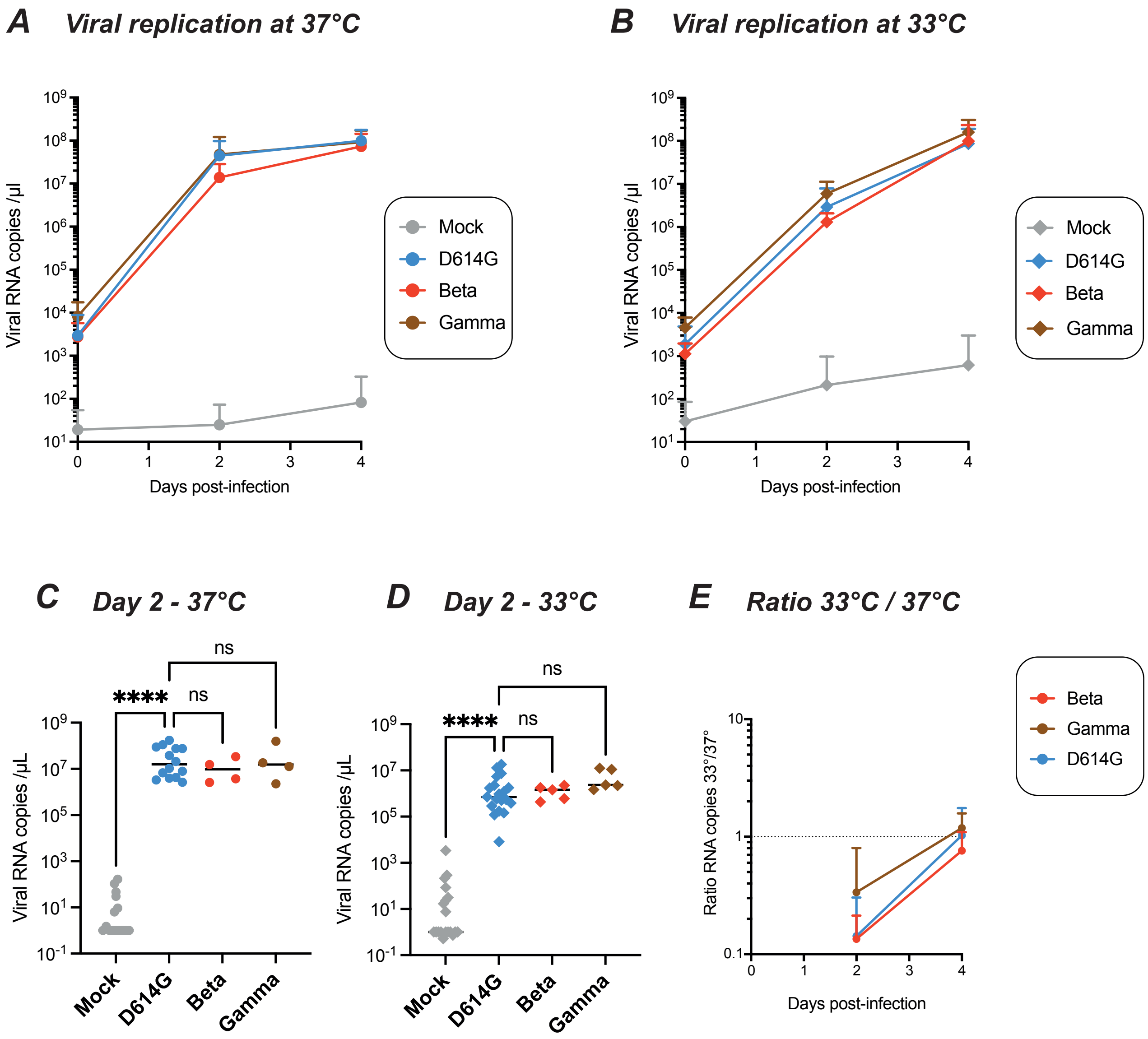

Figure S1: Temperature-dependent replication of the Beta and Gamma SARS-CoV-2 variants in reconstructed human nasal epithelia.

*The viral load in apical supernatants was quantified by RT-qPCR for samples infected by the following SARS-CoV-2 lineages: D614G, Beta, and Gamma. Infections were performed at an input equivalent to 10E8 viral RNA copies. (A, B) Kinetics of viral replication at 37°C (A) and at 33°C (B), with means and SD reported (n=4 to 14 independent samples per point). (C,D) Comparison of apical viral loads measured at 2 days post infection (dpi) at 37°C (C) and 33°C (D). Medians are reported. (E) The ratio of viral RNA copies measured at 33°C to those measured at 37°C is shown at different dpi, with means and SD reported (n=4 to 14 independent samples per point).(C, D, E) Statistical comparisons were done between the D614G reference and all other variants or the Mock condition, using the Kruskal-Wallis test with Dunn's correction; ns: not significant; **** P<0.0001.*
