# Supplementary material for: Stealth replication of SARS-CoV-2 Omicron in the nasal epithelium at physiological temperature
Source: J Virol. 2025 Dec 19;100(1):e02008-25. doi: 10.1128/jvi.02008-25 (PMC12817898; doi:10.1128/jvi.02008-25)
Supplement: Fig. S2 — Infections on nasal epithelia derived from a single donor. [file jvi.02008-25-s0002.pdf]

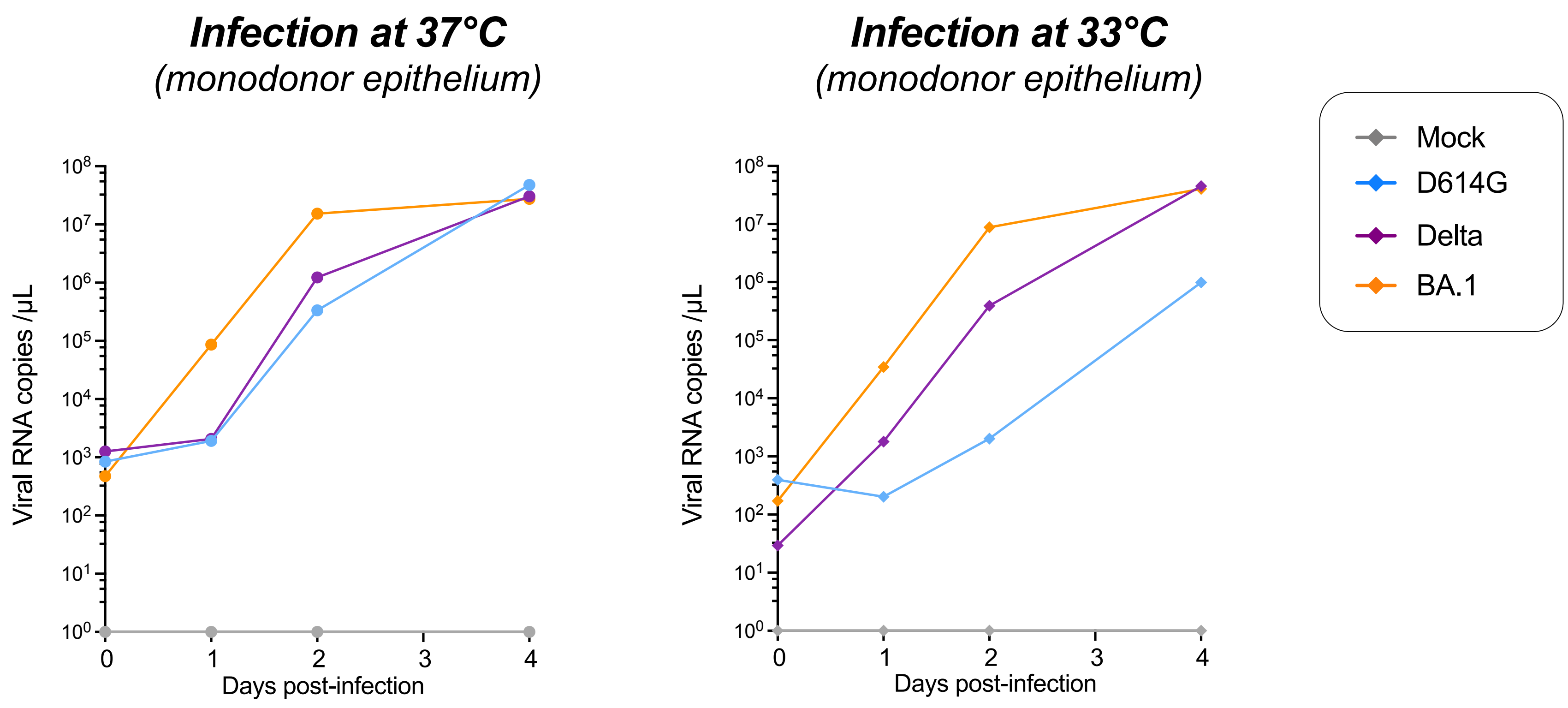

Figure S2: SARS-CoV-2 variant replication in a reconstructed human nasal epithelium generated from a single donor

Reconstructed nasal epithelium samples (MucilAirTM) generated from a single human donor were used in this experiment. Infections were performed at an input equivalent to 10E8 viral RNA copies. The viral load in apical supernatants was quantified by RT-qPCR for epithelial samples left mock-infected or infected by the D614G, Delta and Omicron BA.1. The kinetics of viral replication at 37°C (left) and at 33°C (right) is reported.
