# Supplementary material for: Stealth replication of SARS-CoV-2 Omicron in the nasal epithelium at physiological temperature
Source: J Virol. 2025 Dec 19;100(1):e02008-25. doi: 10.1128/jvi.02008-25 (PMC12817898; doi:10.1128/jvi.02008-25)
Supplement: Fig. S3 — Viral infectivity analysis. [file jvi.02008-25-s0003.pdf]

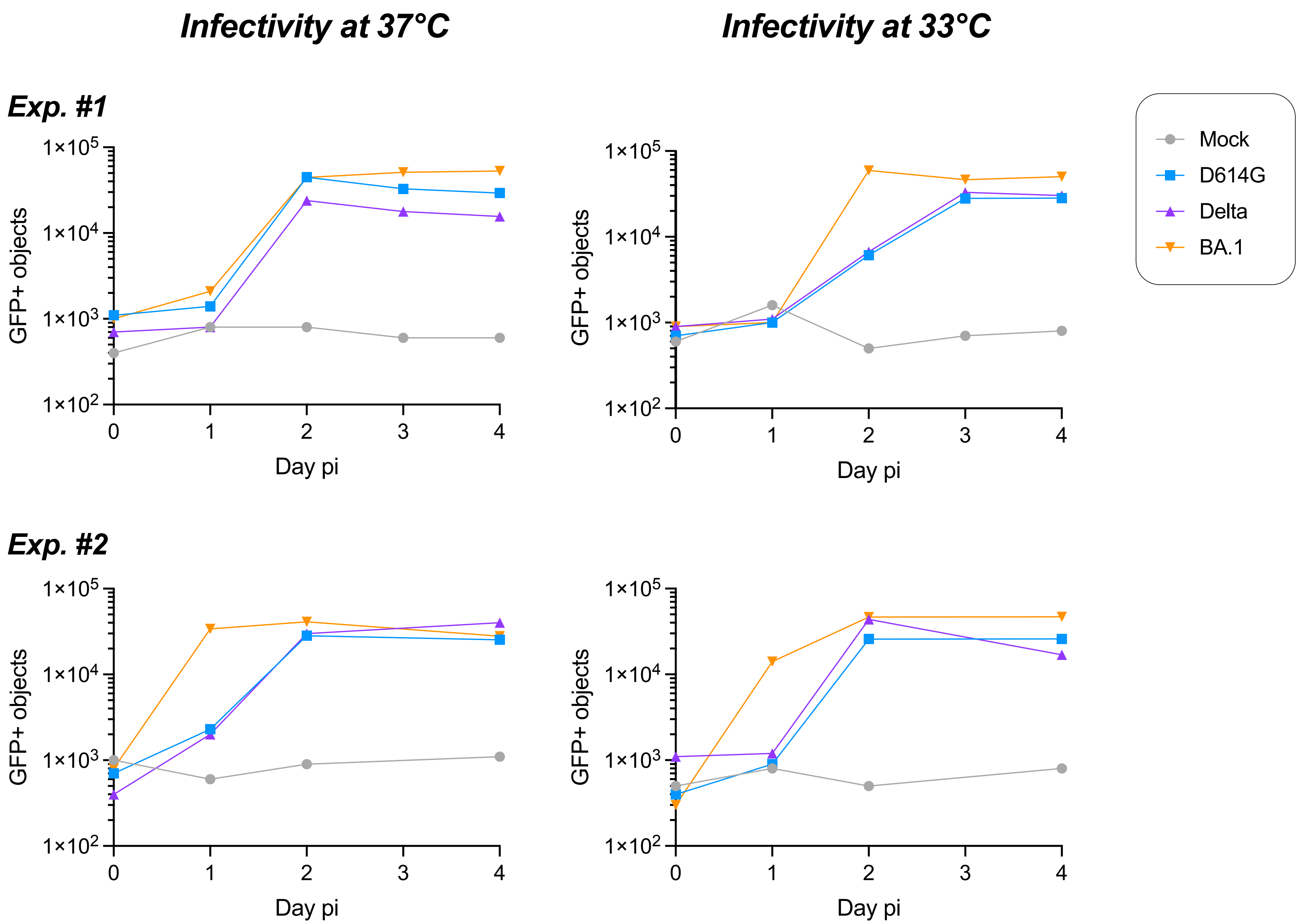

Figure S3: Kinetics of SARS-CoV-2 variant replication measured by the S-fuse infectivity assay

Reconstructed nasal epithelia were infected at 37°C (left) and 33°C (right) in two independent experiments (top and bottom rows, respectively). Infections were performed at an input equivalent to 10E8 viral RNA copies. Viral infectivity in culture supernatants was quantified by automated imaging of cell-cell fusion in a U2OS-ACE2 GFP-split cell system. Infectivity was measured by the number of GFP+ objects per well detected by automated confocal image analysis. pi: post-infection.
