# Supplementary material for: Stealth replication of SARS-CoV-2 Omicron in the nasal epithelium at physiological temperature
Source: J Virol. 2025 Dec 19;100(1):e02008-25. doi: 10.1128/jvi.02008-25 (PMC12817898; doi:10.1128/jvi.02008-25)
Supplement: Fig. S4 — Kinetics of LDH release. [file jvi.02008-25-s0004.pdf]

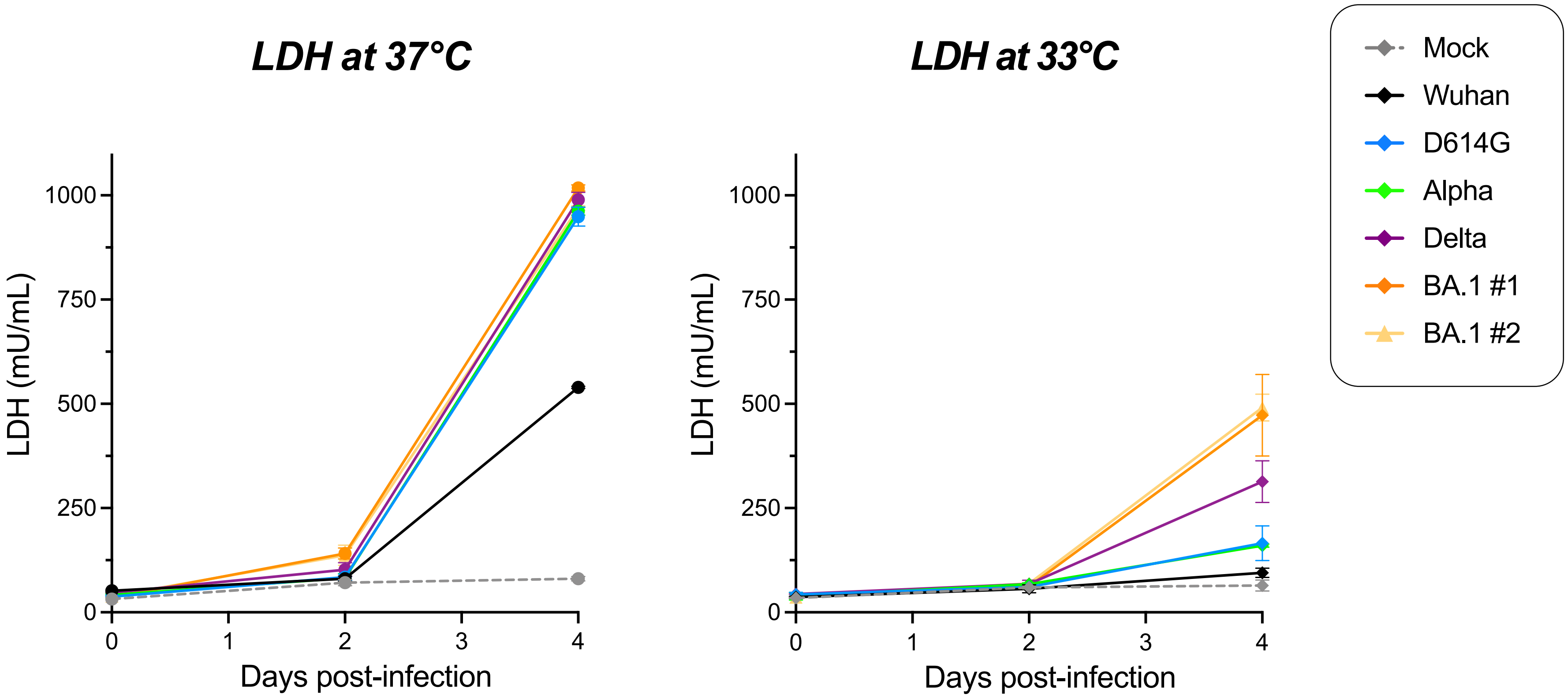

Figure S4: Kinetics of nasal epithelial damage measured by lactate dehydrogenase release
The cytopathic effect induced by SARS-CoV-2 variant infection was assessed at days 0, 2, and 4 post-infection by measuring the release of lactate dehydrogenase (LDH) at 37°C (left) and 33°C (right). Infections were performed at an input equivalent to 10E8 viral RNA copies. Each data point represents the mean of n=2 biological replicates, except for D0 at 37°C, where n=1. Means and SD are shown. Infection by two distinct Omicron BA.1 viral stocks (#1 and #2) was tested in these experiments.
