# Supplementary material for: Stealth replication of SARS-CoV-2 Omicron in the nasal epithelium at physiological temperature
Source: J Virol. 2025 Dec 19;100(1):e02008-25. doi: 10.1128/jvi.02008-25 (PMC12817898; doi:10.1128/jvi.02008-25)
Supplement: Fig. S5 — Scanning electron micrographs of deciliated cells. [file jvi.02008-25-s0005.pdf]

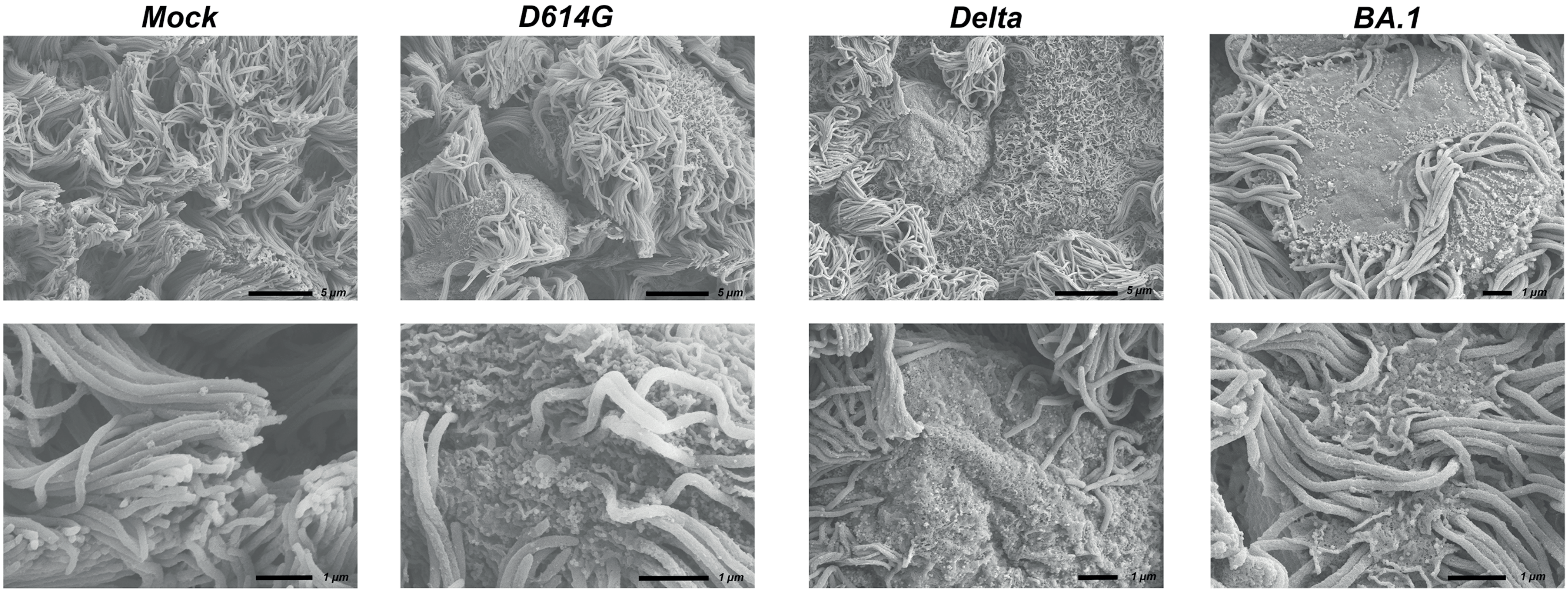

Figure S5: Partially deciliated cells produce viral particles after day 4 of infection at 33°C
Scanning electron microscopy images of reconstructed nasal epithelia at 4 days post-infection. The epithelia were infected at 33°C with the D614G, Delta or Omicron, BA.1 variant (shown in columns 2, 3, 4, respectively) or were mock-infected (first column). Images document partial deciliation upon infection (top row) and the presence of numerous viral particles at the surface of partially deciliated cells (bottom row). The scale bar represents 5 µm or 1 µm, as reported.
