# Supplementary material for: Stealth replication of SARS-CoV-2 Omicron in the nasal epithelium at physiological temperature
Source: J Virol. 2025 Dec 19;100(1):e02008-25. doi: 10.1128/jvi.02008-25 (PMC12817898; doi:10.1128/jvi.02008-25)
Supplement: Fig. S6 — Gene enrichment analysis. [file jvi.02008-25-s0006.pdf]

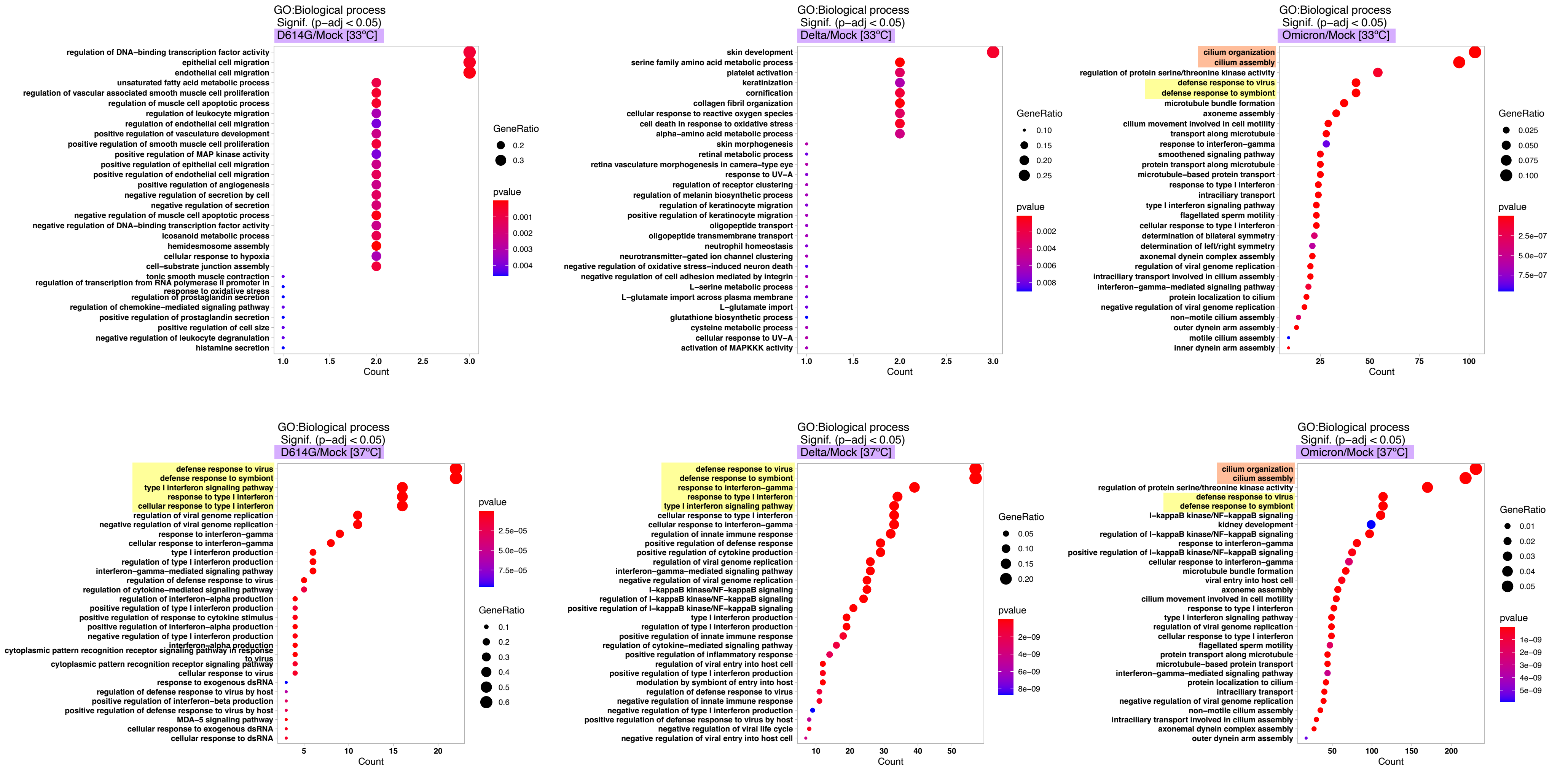

Figure S6: Functional enrichment analysis of transcriptomes from reconstructed nasal epithelia at day 2 post-infection. *The significantly dysregulated gene ontology (GO) terms are reported for epithelial samples infected at 33°C (top) or 37°C (bottom) with the variants D614G (left), delta (middle), and Omicron BA.1 (right). The number of differentially expressed genes (DEGs) belonging to each GO term is reported on the x axis. The size and color of symbols correspond to the gene ratio and p value for each GO term, respectively. Among the 5 top dysregulated GO terms, those corresponding to "cilium organization" and "defense response" are highlighted in orange and yellow, respectively.*
