# Supplementary material for: Stealth replication of SARS-CoV-2 Omicron in the nasal epithelium at physiological temperature
Source: J Virol. 2025 Dec 19;100(1):e02008-25. doi: 10.1128/jvi.02008-25 (PMC12817898; doi:10.1128/jvi.02008-25)
Supplement: Fig. S7 — Volcano plot analysis. [file jvi.02008-25-s0007.pdf]

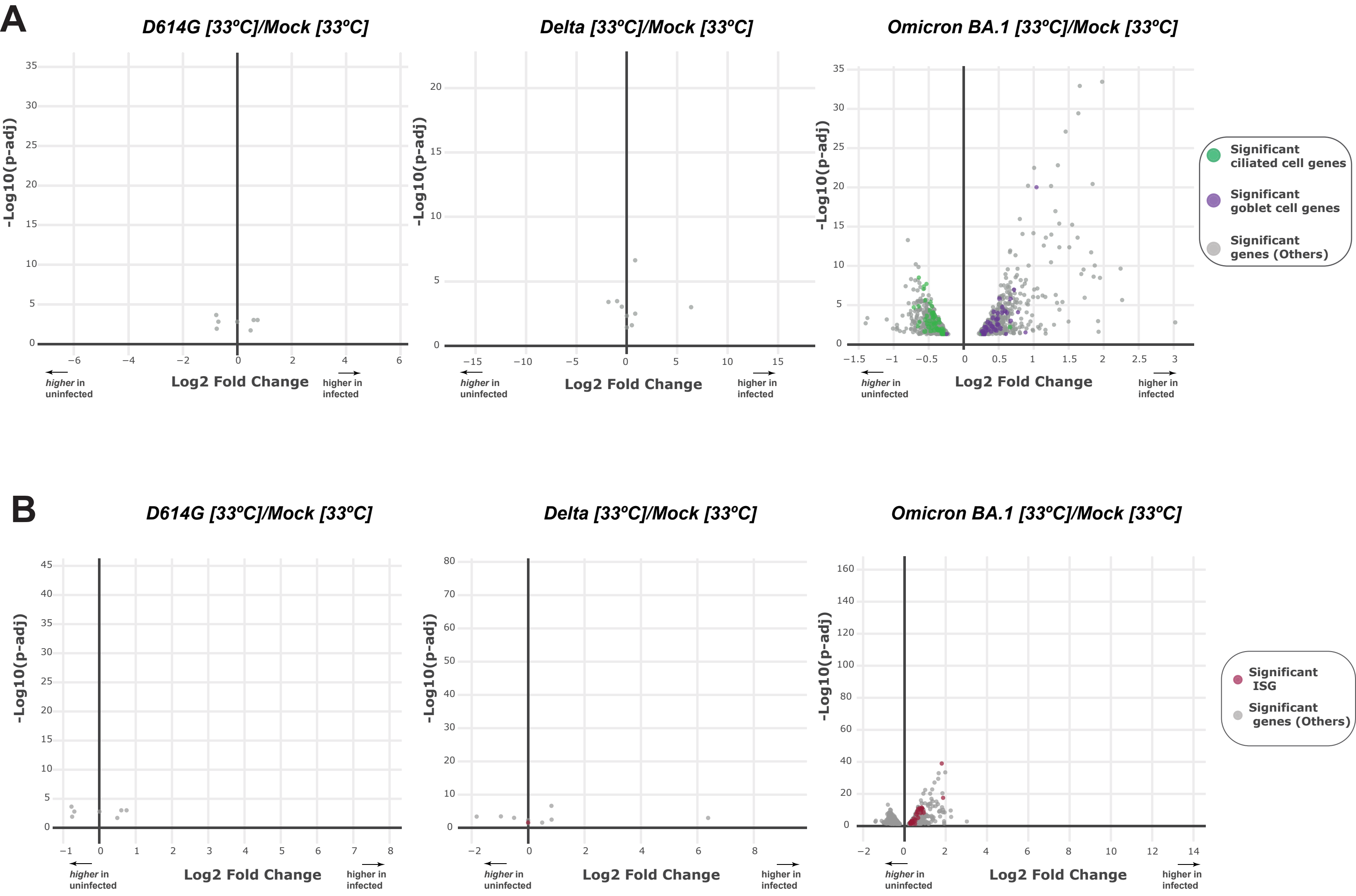

Figure S7: Limited changes in transcriptional profiles of reconstructed nasal epithelia infected at 33°C.
(A) Volcano-plots depicting the differential regulation of ciliated cell genes (green) and goblet cell genes (purple) at 33°C in variant-infected samples compared to mock-infected samples at 2 dpi. The log2 fold change in gene expression is shown on the x axis and the adjusted p value on the y axis. Genes overexpressed in infected samples are distributed to the right on the x axis. Significant DEGs that do not belong to the ciliated cell or goblet cell category are represented in grey. (B) Volcano-plots depicting the differential regulation of ISGs (red) at 33°C in variant-infected samples compared to mock-infected samples at 2 dpi. The representation is similar to that in (A).
