# Supplementary material for: Stealth replication of SARS-CoV-2 Omicron in the nasal epithelium at physiological temperature
Source: J Virol. 2025 Dec 19;100(1):e02008-25. doi: 10.1128/jvi.02008-25 (PMC12817898; doi:10.1128/jvi.02008-25)
Supplement: Table S1 — Information on epithelial cell donors. [file jvi.02008-25-s0009.pdf]

Supplementary Table 1: Information on pools of donors used to generate MucilAirTM reconstructed nasal epithelia

	MP0009 Pool				MP0010 Pool				MP0011 Pool		
	Donor ID	Age	Sex		Donor ID	Age	Sex		Donor ID	Age	Sex
Donor 1	813	52	M		870	77	F		887	39	F
Donor 2	809	24	M		866	54	M		892	36	M
Donor 3	808	36	M		863	57	F		881	47	F
Donor 4	806	32	M		825	65	F		817	40	M
Donor 5	803	58	M		824	69	M		635	36	M
Donor 6	798	44	F		809	24	M		605	65	M
Donor 7	797	55	M		807	61	M		471	40	F
Donor 8	780	55	M		794	53	M		874	53	M
Donor 9	762	N/A	N/A		774	38	M		866	54	M
Donor 10	755	54	F		771	23	M		861	73	M
Donor 11	745	53	M		767	71	M		853	35	M
Donor 12	738	32	F		760	45	N/A		831	27	M
Donor 13	735	57	M		745	45	M		692	58	F
Donor 14	731	58	M		507	29	M		809	24	M

	MP0009 Pool	MP0010 Pool	MP0011 Pool
Median age (yrs)	53	53.5	40
Min age (yrs)	24	23	24
Max age (yrs)	58	77	73

Legend: Reconstructed human nasal epithelia (MucilAirTM) were generated by the Epithelix company (www.epithelix.com)
To limit individual variability, each reconstructed epithelium was grown from biological material derived from a pool of 14 donors.
Epithelia obtained from three distinct pools of donors (MP009, MP0010; and MP0011) were used in the present study.
The ID number, age, and sex (F: female; M: male) of each donor is provided for the 3 pools. N/A: not available.
