# Supplementary material for: Stealth replication of SARS-CoV-2 Omicron in the nasal epithelium at physiological temperature
Source: J Virol. 2025 Dec 19;100(1):e02008-25. doi: 10.1128/jvi.02008-25 (PMC12817898; doi:10.1128/jvi.02008-25)
Supplement: Table S2 — Viral isolates used in the study. [file jvi.02008-25-s0010.pdf]

Supplementary Table 2: Viral Stocks used in the study

Variant of concern	Strain derivation	Gisaid/EVAg	Reference	DOI
**Wuhan**	BetaCoV/France/IDF00372/2020	Ref-SKU: 014V-03890	Robinot R et al, Nat Comm. 2021	doi: 10.1038/s41467-021-24521-x
**D614G**	hCoV-19/France/GE1973/2020*	EPI_ISL_414631	Planas D et al, Nat Med 2021	doi: 10.1038/s41591-021-01318-5
**Alpha (B.1.1.7)**	hCoV-19/France/CVL-SC719/2020	EPI_ISL_735391	Planas D et al, Nat Med 2021	doi: 10.1038/s41591-021-01318-5
**Beta (B.1.351)**	hCoV-19/France/IDF-IPP00078/2021	EPI_ISL_964916	Planas D et al, Nat Med 2021	doi: 10.1038/s41591-021-01318-5
**Gamma (P.1)**	hCoV-19/Japan/TY7-501/2021	EPI_ISL_833366	Betton M. et al., Clin Inf Dis 2021	doi: 10.1093/cid/ciab308
**Delta (B.1.617)**	hCoV-19/France/IDF-APHP-HEGP-20-23-2131905084/2021	EPI_ISL_2029113	Planas D et al, Nature 2021	doi: 10.1038/s41586-021-03777-9
**Omicron (BA.1)**	hCoV-19/Belgium/rega-20174/2021	EPI_ISL_6794907	Planas D et al, Nature 2022	doi: 10.1038/s41586-021-04389-z

Notes *Viral isolate supplied by the National Reference Centre for Respiratory Viruses hosted by Institut Pasteur
