# Supplementary material for: Stealth replication of SARS-CoV-2 Omicron in the nasal epithelium at physiological temperature
Source: J Virol. 2025 Dec 19;100(1):e02008-25. doi: 10.1128/jvi.02008-25 (PMC12817898; doi:10.1128/jvi.02008-25)
Supplement: Fig. S8 — Interferon production at day 4. [file jvi.02008-25-s0008.pdf]

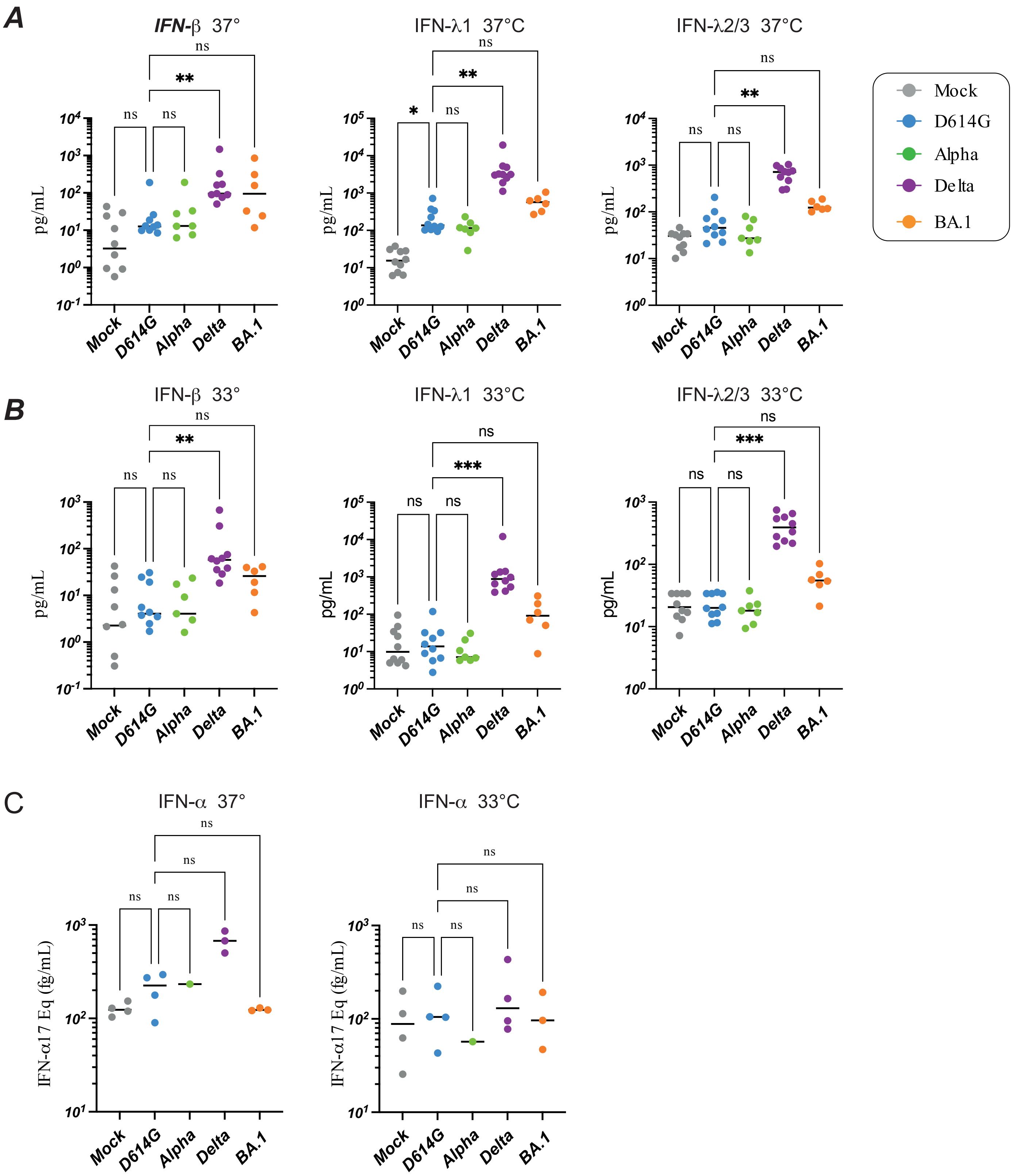

Figure S8: Induction of type I and type III IFNs by SARS-CoV-2 variants at day 4 post-infection.
*(A) The induction of IFN-β (left), IFN-λ1 (middle) and IFN-λ2/3 (right) release in apical supernatants of reconstructed nasal epithelia infected at 37°C was measured by digital ELISA (IFN-β) and Legendplex assays (IFN-λ). (B) Similar graphs as in (A), but for infections carried out at 33°C. (C) The induction of IFN-α release in apical supernatants of reconstructed nasal epithelia infected at 37°C (left) and 33°C (right) was measured by digital ELISA, with results expressed as IFN-α17 equivalent. (A-C) Medians are reported. Statistical comparisons were done between the D614G reference variants and all the other variants tested, using the Kruskal-Wallis test with Dunn's correction; ns: not significant; ns: not significant; * P<0.05; ** P<0.01; *** P<0.001.*
